# Neurotrophins, cytokines, oxidative stress mediators and mood state in bipolar disorder: systematic review and meta-analyses

**DOI:** 10.1192/bjp.2018.144

**Published:** 2018-09

**Authors:** Tobias Rowland, Benjamin I. Perry, Rachel Upthegrove, Nicholas Barnes, Jayanta Chatterjee, Daniel Gallacher, Steven Marwaha

**Affiliations:** 1 IHR Academic Clinical Fellow in Psychiatry, Mental Health and Wellbeing, Warwick Medical School, University of Warwick, UK; 2 NIHR Academic Clinical Fellow in Psychiatry, Mental Health and Wellbeing, Warwick Medical School, University of Warwick, UK; 3 Senior Clinical Lecturer in Psychiatry, Institute of Clinical Sciences, School of Clinical and Experimental Medicine, University of Birmingham, UK; 4 Professor of Neuropharmacology, Institute of Clinical Sciences, School of Clinical and Experimental Medicine, University of Birmingham, UK; 5 Consultant Psychiatrist, Affective Disorders Service, Caludon Centre, Coventry, UK; 6 Research Associate in Medical Statistics, WMS Population, Evidence and Technologies, Warwick Medical School, University of Warwick, UK; 7 Reader in Psychiatry, Mental Health and Wellbeing, Warwick Medical School, University of Warwick, UK

**Keywords:** Biomarkers, bipolar disorder, cytokines, neurotrophins, oxidative stress, meta-analysis, review

## Abstract

**Background:**

A reliable biomarker signature for bipolar disorder sensitive to illness phase would be of considerable clinical benefit. Among circulating blood-derived markers there has been a significant amount of research into inflammatory markers, neurotrophins and oxidative stress markers.

**Aims:**

To synthesise and interpret existing evidence of inflammatory markers, neurotrophins and oxidative stress markers in bipolar disorder focusing on the mood phase of illness.

**Method:**

Following PRISMA (Preferred Reporting Items for Systematic reviews and Meta-analyses) guidelines, a systematic review was conducted for studies investigating peripheral biomarkers in bipolar disorder compared with healthy controls. We searched Medline, Embase, PsycINFO, SciELO and Web of Science, and separated studies by bipolar mood phase (mania, depression and euthymia). Extracted data on each biomarker in separate mood phases were synthesised using random-effects model meta-analyses.

**Results:**

In total, 53 studies were included, comprising 2467 cases and 2360 controls. Fourteen biomarkers were identified from meta-analyses of three or more studies. No biomarker differentiated mood phase in bipolar disorder individually. Biomarker meta-analyses suggest a combination of high-sensitivity C-reactive protein/interleukin-6, brain derived neurotrophic factor/tumour necrosis factor (TNF)-α and soluble TNF-α receptor 1 can differentiate specific mood phase in bipolar disorder. Several other biomarkers of interest were identified.

**Conclusions:**

Combining biomarker results could differentiate individuals with bipolar disorder from healthy controls and indicate a specific mood-phase signature. Future research should seek to test these combinations of biomarkers in longitudinal studies.

**Declaration of interest:**

None.

Bipolar affective disorder (bipolar disorder) is a relatively common severe mental illness, with worldwide lifetime prevalence of 2.4%,^1^ and ranks among the top ten causes of disability worldwide.^2^ Currently, diagnosis is based on clinical interview, and in the absence of biological markers this process has been criticised as lacking objectivity and having poor reliability and validity.^3^ Indeed, initial diagnosis and correct treatment are often delayed by 6–10 years,^4^ in part because of the difficulties of making a clinical diagnosis. This can have a considerable impact upon clinical outcome given that earlier treatment is more effective.^5^ Circulating blood-derived biomarkers represent potential objective tests that may help to address this clinical need and there has been a surge in relevant research in recent years, particularly within the categories of neurotrophins, inflammatory markers and oxidative stress markers. Moreover, the phase of bipolar illness appears to be related to some biomarker levels,^6^^,^^7^ and therefore it is necessary to analyse biomarkers according to mood phase.

Neurotrophins are mediators in the stress response and promote neuronal well-being, influencing neuronal survival, cell proliferation, plasticity^8^^,^^9^ and long-term memory.^10^ The majority of studies on neurotrophins in bipolar disorder have focused on brain derived neurotrophic factor (BDNF). Six meta-analyses of this data^7^^,^^11^^–^^14^ have found significantly lower levels of BDNF in bipolar disorder compared with healthy controls and people with unipolar depression, and lowered BDNF in bipolar mania and depression.^14^ Neuroinflammation within the central nervous system and neuro-humoral pathways (for example the hypothalamic–pituitary–adrenal axis) is linked to mood disorders.^15^ Cytokines such as interleukins (ILs), namely IL-2, IL-4, IL-6 and tumour necrosis factor alpha (TNF-α) together with the inflammatory marker c-reactive protein (CRP) have been identified as potentially relevant blood-borne biomarkers in bipolar, and may change in different affective states.^6^^,^^16^^,^^17^ The development of the oxidative stress theory of bipolar disorder led to identification of several potential biomarkers such as nitric oxide (NO), thiobarbituric acid reactive substances (TBARS) and lipid peroxidase (LPO). Meta-analyses have found that levels of TBARS, NO and LPO are significantly raised in people with bipolar disorder compared with healthy controls, although participants were not separated by mood phase.^18^^,^^19^

In the existing literature, syntheses to date have indicated an effect of mood phase on biomarker levels,^6^^,^^7^ but have focused on individual markers or are limited by the small number of studies investigating each mood phase. There is increasing interest in the use of biomarker combinations as diagnostic panels.^20^^,^^21^ These have been used in many branches of medicine, such as thyroid disorders^22^ and in the ‘triple test’ for Down syndrome in pregnancy.^23^ As yet, this is an underutilised paradigm within psychiatric disorders. This systematic review and the meta-analyses therefore aimed to synthesise and interpret existing evidence and present aggregated effect with focus on biomarkers by phase of illness in bipolar disorder. Our objective was to provide preliminary evidence and inform research strategies to deliver selective biomarker signatures for bipolar disorder. As such this evidence synthesis is designed to be exploratory, as opposed to testing particular hypotheses.

## Method

Following PRISMA (Preferred Reporting Items for Systematic reviews and Meta-analyses) guidelines,^24^ a systematic review and meta-analyses were conducted for circulating biomarkers in blood or serum of patients with bipolar disorder compared with healthy controls. An initial scoping review was conducted to identify potential biomarkers in bipolar disorder to generate a list of search terms.

We searched Embase (1947–present), Ovid Medline (1946–present), Web of Science (inception–present) SciELO (1998–present) and PsycINFO (1806–present) to 1 February 2017. The first 20 pages of Google Scholar were also searched as recommended in a recent review,^25^ alongside searching references of included studies for search keywords. The following MeSH headings or their equivalent and text terms were used: bipolar disorder, bipolar, ‘antidepressant induced’, mania, manic, depression, depressive, depressive syndrome, euthymia, euthymic grouped with biomarker, predictor, blood, neurotrophin, oxidative stress, brain derived neurotrophic factor, BDNF, cytokine, monoamine, dopamine, homovanillic acid, HVA, interleukin, tumour necrosis factor, TNF, C reactive protein, CRP, TBARS, 3-NT, NO,

Inclusion criteria were:

(a) primary studies evaluating biomarker levels in patients with bipolar disorder (in any mood phase);
(b) bipolar disorder diagnosis confirmed using structured clinical instruments;
(c) patients aged >16 years;
(d) matched healthy control group;
(e) studies in any language;
(f) peer-reviewed papers, alongside searchable conference abstracts and theses;
(g) methods for biomarker assay were validated commercially available assays.

Exclusion criteria were:

(a) studies focusing purely on genetic differences between groups (no measured biomarker);
(b) studies including participants under the age of 16 either in the patient or control group as rates of bipolar disorder are relatively rare below this age, hence diagnostic accuracy in these patients might be reduced

### Study selection

Study titles and/or abstracts were screened independently by two authors (B.P., J.C.) applying the inclusion criteria, in order to decide on studies that would be examined in full-text form. Any discrepancies were resolved in consultation with the senior author (S.M.).

Full-text studies were examined independently by two review authors (B.P., J.C.) for final inclusion. Risk of bias was assessed by two authors independently (B.P. and T.R.) using the Newcastle–Ottawa Scale for non-randomised studies.^26^ Disagreement over risk of bias assessment was resolved through involvement of a third senior review author (S.M.). Data were extracted by three reviewers (B.P., J.C. and T.R.). Details included participant characteristics, diagnostic criteria, study design, outcomes measured and data for analysis.

In studies that did not report data from which the mean and standard deviation (s.d.) could be calculated, the corresponding author was contacted to request the original data. If no response was received, or data were unavailable these studies were not included in the meta-analysis but were discussed narratively.

### Analysis

The mean and s.d.s for each biomarker, separated into mood phase, were subject to meta-analysis using RevMan 5.3, with mean differences and 95% CIs between cases and controls calculated and displayed in forest plots. The inverse variance method was used where the weight given to each study is the inverse of the variance of the effect estimate. Random-effects models were used to pool data because of substantial clinical and methodological heterogeneity between studies. A meta-analysis was completed if there were two or more separate samples of the same mood phase measuring a given biomarker, although only meta-analyses of three or more studies are used for drawing conclusions and identifying discriminatory biomarkers. A separate sensitivity analysis using only studies sampling patients who were psychotropic medication free (psychotropics defined as antipsychotics, antidepressants, mood stabilisers, hypnotics, anxiolytics) was completed. Publication bias was assessed by use of funnel plots that were assessed for asymmetry in each meta-analysis including ten or more studies, as is recommended for interpretation.^27^^,^^28^

### Defining discriminatory biomarkers

We propose that a ‘fully discriminatory’ biomarker for bipolar disorder should be significantly different compared with that found in matched healthy controls in only one mood phase of bipolar disorder, to allow it to discriminate that mood phase. A ‘partially discriminatory’ biomarker should be significantly different from that found in healthy controls in two mood phases of bipolar disorder, allowing it to discriminate the mood phase in which it is not altered from controls.

## Results

**Fig. 1:**
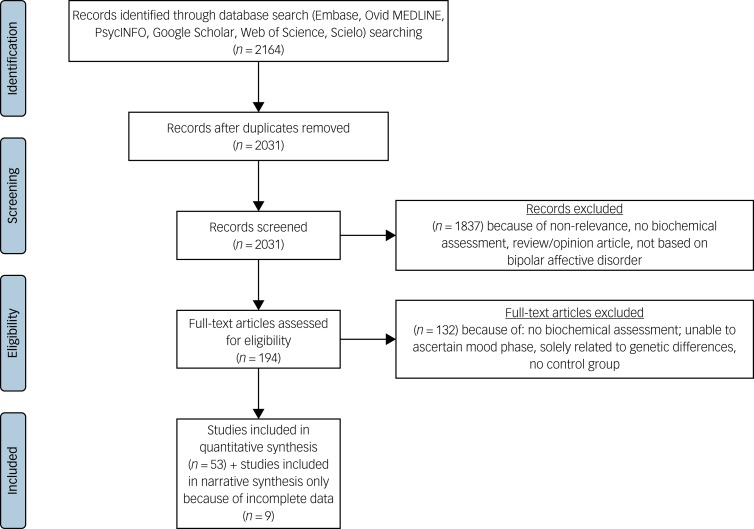
PRISMA (Preferred Reporting Items for Systematic reviews and Meta-analyses) diagram.

The search strategy retrieved 6348 studies; 194 full texts were reviewed, and, of these, 62 studies met inclusion criteria.^29^^–^^90^ Thirteen of these studies did not provide data from which the mean and s.d. could be calculated, and the corresponding authors were contacted. Nine studies were unable to provide this data, so were excluded from the meta-analysis but their results are discussed narratively. Subsequently 53 were included in the quantitative synthesis (Fig. 1). Because of the number of biomarkers (*n* = 14), and our objective to understand their mood phase specificity it was necessary to complete 31 meta-analyses.

### Characteristics of the available literature

Supplementary Table 1 (available at https://doi.org/10.1192/bjp.2018.144) outlines the characteristics of the included studies. Of the 53 studies included in meta-analyses, 49 were of case–control design, 3 were prospective cohort studies^50^^,^^58^^,^^65^ and one was a non-randomised trial.^64^ Baseline data were extracted, and these were treated as case–control studies.

A total of 37 studies investigated patients in a manic or hypomanic phase, 40 investigated patients in a euthymic mood state and 26 investigated depression. The determination of mood phase varied significantly between studies, and the majority used the Young's Mania Rating Scale (YMRS) and Hamilton Rating Scale for Depression (HRSD) to assess symptom severity. The most common biomarker assay used was commercial enzyme-linked immunosorbent assay kits, and the specific rating scales, cut-off points and assays are described in supplementary Table 1.

### Participant and clinical features of samples in included studies

The 53 studies included in the meta-analyses included 2467 case participants and 2360 controls, with the number of case participants in each study varying from 10 to 141. There was a slight female predominance overall (55.6%). The mean age across studies was 38.7 years calculated where age data was available, and the mean age within studies ranged from 23 to 65 years. Specific diagnostic criteria, interview tools and main exclusion criteria of studies are detailed in supplementary Table 1. A total of 33 studies specified a diagnosis of bipolar disorder type I, a further 7 studies also included bipolar disorder type II and 1 study investigated patients with rapid-cycling bipolar disorder, including both type I and II bipolar disorder.^65^

Most (*n* = 32) studies explicitly stated that controls were age matched, and 30 also matched for gender. All studies specified controls were free from other psychiatric illnesses, with most having used the Structured Clinical Interview for DSM-IV for this purpose.

Of the case–control studies, 15 followed up participants before and after treatment.^30^^,^^46^^,^^52^^,^^54^^–^^56^^,^^59^^,^^61^^,^^71^^,^^74^^–^^77^^,^^79^ For the purpose of the meta-analyses the values for biomarkers after treatment were only included where the study had explicitly stated participants were in remission following treatment and had defined the mood phase objectively, and these were analysed as separate case–control studies.^52^^,^^60^^,^^75^^–^^77^^,^^79^

### Medication status

Most studies included patients taking medications (for example lithium, anticonvulsants, antipsychotics, antidepressants, benzodiazepines), several of which can affect oxidative stress and cytokine levels.^91^^–^^94^ Fourteen studies explored patients entirely free from medication for up to 8 weeks prior to the study. These studies also included some patients who were drug naive, but only one study reported only participants who were completely medication naive.^58^ Three studies included patients receiving electroconvulsive therapy.^52^^,^^71^^,^^74^

### Risk of bias in included studies

Studies were appraised using the Newcastle–Ottawa Scale,^26^ treating each study as a case–control study for the purpose of the meta-analysis. The results are shown in supplementary Table 2. Studies scored between two and eight of a possible nine, and there was significant variability between studies in all three sections of the assessment. Funnel plots were examined for each meta-analysis with more than ten studies (details available on request from the authors), but were not found to be clearly asymmetrical, although most analyses fell below this threshold. However, this is in keeping with the general findings of the study, which included findings of many publications of negative results for biomarkers.

### Study results

We present a summary of the findings of our meta-analyses in Table 1, which includes the number of studies/participants, the corresponding summary effect size and *I*^2^ value for each meta-analysis. Effect sizes >0.8 were regarded as ‘large’ and >0.5 as ‘moderate’.^95^ Forest plots are included for biomarkers that were analysed in each mood phase (Fig. 2–4), and forest plots for the remaining biomarkers are included in supplementary Figs 2–24. Several biomarkers of distinct categories, including neuroendocrine markers, were identified but insufficient studies were available in each mood phase to conduct meta-analyses (see supplementary Table 1).

**Table 1:** Discriminatory and potentially discriminatory biomarkers

Biomarker	Depression			Euthymia			Mania		
	Studies/ participants, *n*	Result	Effect size (95% CI), *P* (I_2_, %)	Studies/ participants, *n*	Result	Effect size (95% CI), *P* (I_2_)	Studies/ participants, *n*	Result	Effect size (95% CI), *P* (I_2_, %)
Discriminatory biomarkers in combination									
BDNF	13/949	↓	−0.86 (−1.38 to −0.34) 0.001 (91)	14/1447	↔	0.12 (−0.11 to 0.35) 0.32 (77)	13/946	↓	−0.54 (−1.03 to −0.06) 0.03 (91)
hsCRP	3/420	↔	−0.02 (−0.25 to 0.21) 0.86 (0)	4/547	↑	0.8 (0.3 to 1.3) 0.002 (81)	6/640	↑	0.74 (0.37 to 1.11) <0.0001 (71)
IL6	6/601	↔	0.67 (−0.08 to 1.42) 0.08 (91)	6/734	↑	0.71 (0.07 to 1.34) 0.03 (92)	6/695	↑	1.07 (0.29 to 1.84) 0.007 (93)
sTNFR1	3/479	↔	0.46 (−0.02 to 0.94) 0.06 (62)	7/727	↔	0.39 (−0.21 to 1.00) 0.21 (91)	5/609	↑	0.96 (0.43 to 1.49) 0.0004 (82)
TNF-α	6/334	↑	2.09 (0.82 to 3.36) 0.001 (94)	8/522	↔	0.33 (−0.13 to 0.79) 0.16 (82)	7/515	↑	1.74 (0.88 to 2.59) <0.0001 (93)
Potential discriminatory biomarkers									
IL-1RA	<3^a^	–	–	5/469	↔	0.33 (−0.03 to 0.69) 0.07 (56)	4/399	↑	0.45 (0.03 to 0.88) 0.03 (53)
sIL-2R	<3^a^	–	–	3/313	↑	0.86 (0.29 to 1.44) 0.003 (78)	3/279	↑	1.06 (0.66 to 1.46) <0.0001 (49)
sIL-6R	<3^a^	–	–	4/359	↑	0.61 (0.25 to 0.97) 0.001 (56)	3/279	↑	0.34 (0.08 to 0.60) 0.01 (0)
Biomarkers that do not appear to be altered									
IL-2	<3^a^	–	–	3/158	↔	0.46 (−0.16 to 1.08) 0.15 (65)	3/224	↔	−0.83 (−2.3 to 0.65) 0.27 (95)
IL4	<3^a^	–	–	4/221	↔	0.23 (−0.11 to 0.56) 0.18 (38)	6/474	↔	0.93 (−0.11 to 1.97) 0.08 (96)
IL10	<3^a^	–	–	6/378	↔	0.23 (−0.08 to 0.55) 0.15 (54)	4/250	↔	0.24 (−0.03 to 0.52) 0.08 (0)
IFN-γ	<3^a^	–	–	5/263	↔	0.03 (−0.64 to 0.7) 0.92 (86)	5/427	↔	0.19 (−0.37 to 0.75) 0.51 (86)

BDNF, brain derived neurotrophic factor; ↓, lower in case participants; ↔ , not significantly different from controls; hsCRP, high-sensitivity C-reactive protein; ↑, higher in case participants; IL, interleukin; sTNFR1, soluble tumour necrosis factor (TNF)-α receptor 1; sIL-2R, soluble IL-2 receptor; IFN, interferon.

a. Fewer than three studies therefore analysis not carried out.

### Inflammatory mediators

**Fig. 2:**
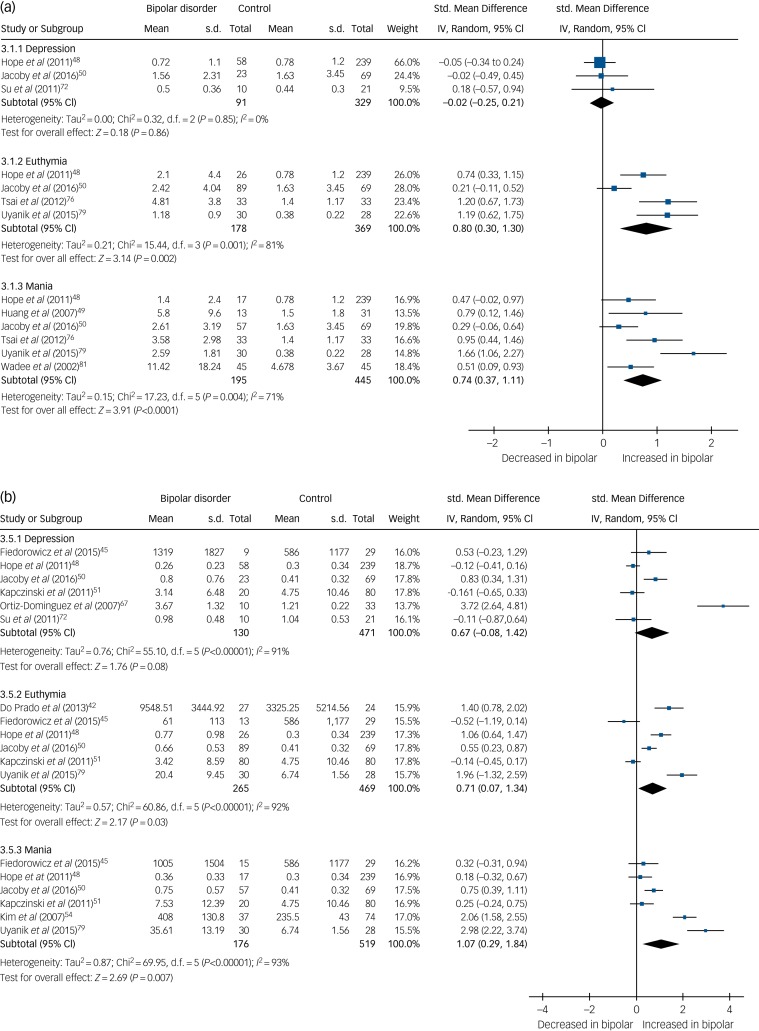
(a) Forest plot of high-sensitivity C-reactive protein (hsCRP) in depression, euthymia and mania compared with healthy controls and (b) forest plot of interleukin (IL)-6 in depression, euthymia and mania compared with healthy controls. Std, standard.

In total, 32 studies investigated levels of inflammatory mediators. High-sensitivity CRP (hsCRP) was found to be elevated in both euthymia (standardised mean difference (SMD) 0.8, 95% CI 0.3 to 1.3, *P* = 0.002, *I*^2^ = 81%, number of studies (*k*) = 4, number of participants (*n*) = 547) and mania (SMD 0.74, 95% CI 0.37 to 1.11, *P*<0.0001, *I*^2^ = 71%, *k* = 6, *n* = 640), but in bipolar depression there was no significant difference from controls (SMD −0.02, 95% CI −0.25 to 0.21, *P* = 0.86, *I*^2^ = 0%, *k* = 3, *n* = 420) (Fig. 2(a)).

IL1-RA levels were found to be elevated in mania (SMD 0.45, 95% CI 0.03 to 0.88, *P* = 0.03, *I*^2^ = 53%, *k* = 4, *n* = 399), but not significantly raised in euthymia (SMD 0.33, 95% CI −0.03 to 0.69, *P* = 0.07, *I*^2^ = 56%, *k* = 5, *n* = 469). Only two studies examined IL1-RA in bipolar depression, but levels were not significantly different from controls (supplementary Fig. 2).

IL-6 was found to be increased in mania (SMD 1.07, 95% CI 0.29 to 1.84, *P* = 0.007, *I*^2^ = 93%, *k* = 6, *n* = 695) and euthymia (SMD 0.71, 95% CI 0.07 to 1.34, *P* = 0.03, *I*^2^ = 92%, *k* = 6, *n* = 734) but not significantly elevated in bipolar depression (SMD 0.67, 95% CI −0.08 to 1.42, *P* = 0.08, *I*^2^ = 91%, *k* = 6, *n* = 601) (Fig. 2(b)).

Soluble IL-2 receptor (sIL-2R) were increased in both euthymia (SMD 0.86, 95% CI 0.29–1.44, *P* = 0.003, *I*^2^ = 78%, *k* = 3, *n* = 313) and mania (SMD 1.06, 95% CI 0.66–1.46, *P* < 0.00001, *I*^2^ = 49%, *k* = 3, *n* = 279) (supplementary Fig. 3). There were insufficient studies to perform a meta-analysis for patients with bipolar depression, but one study^32^ reported increased levels in bipolar depression (*P* < 0.0001), and another^83^ reported increased levels across all mood phases (*P* < 0.01).

Soluble IL-6 receptor (sIL-6R) was increased in mania (SMD 0.34, 95% CI 0.08–0.6, *P* = 0.01, *I*^2^ = 0%, *k* = 3, *n* = 279) and increased in euthymia (SMD 0.61, 95% CI 0.25–0.97, *P* = 0.001, *I*^2^ = 56%, *k* = 4, *n* = 359) (supplementary Fig. 4). Insufficient studies were available to conduct a meta-analysis for bipolar depression, but one study^32^ found sIL-6R levels were significantly increased in bipolar depression (*P* < 0.0001).

**Fig. 3:**
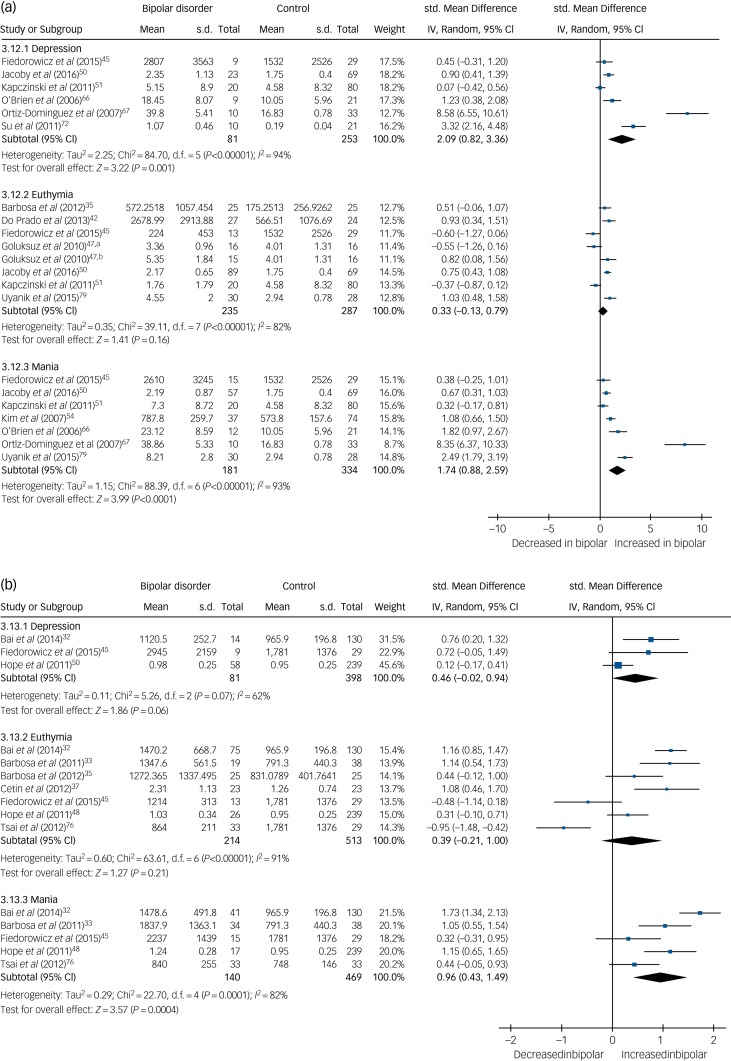
(a) Forest plot of tumour necrosis factor (TNF)-α in depression, euthymia and mania compared with healthy controls and (b) forest plot of soluble TNF-α receptor 1 (sTNFR1) in depression, euthymia and mania compared with healthy controls. a. Medication free; b. lithium monotherapy. Std, standard.

TNF-α levels were significantly increased in both mania (SMD 1.74, 95% CI 0.88 to 2.59, *P* < 0.0001, *I*^2^ = 93%, *k* = 7, *n* = 515) and bipolar depression (SMD 2.09, 95% CI 0.82 to 3.36, *P* < 0.001, *I*^2^ = 94%, *k* = 6, *n* = 334) but not significantly different in euthymia (SMD 0.33, 95% CI −0.13 to 0.79, *P* = 0.16, *I*^2^ = 82%, *k* = 8, *n* =  522) (Fig. 3(a)). One small study^67^ led to the particularly large effect sizes in both mania and bipolar depression, and a sensitivity analysis excluding that study reduced the effect size, although the results remained significant and the effect size remained large (SMD >0.8). A further four studies assessed TNF-α levels but were not included in the meta-analysis, shown in supplementary Table 1.

Soluble TNF-α receptor 1 (sTNFR1) levels were found to be increased in mania (SMD 0.96, 95% CI 0.43 to 1.49, *P* = 0.0004, *I*^2^ = 82%, *k* = 5, *n* = 609) but not significantly different in euthymia (SMD 0.39, 95% CI −0.21 to 1.0, *P* = 0.21, *I*^2^ = 91%, *k* = 7, *n* = 727) or bipolar depression (SMD 0.46, 95% CI −0.02 to 0.94, *P* = 0.06, *I*^2^ = 62%, *k* = 3, *n* = 479) (Fig. 3(b)).

IL-2, IL-4, IL-10 and interferon (IFN)-γ levels were not found to be different from controls in mania or euthymia (supplementary Figs 5–8). There were too few studies to conduct meta-analyses for bipolar depression for these biomarkers. Individual studies did not find significant differences from controls. For the remaining lammatory markers there were too few studies to conduct meaningful meta-analyses.

### Neurotrophins

**Fig. 4:**
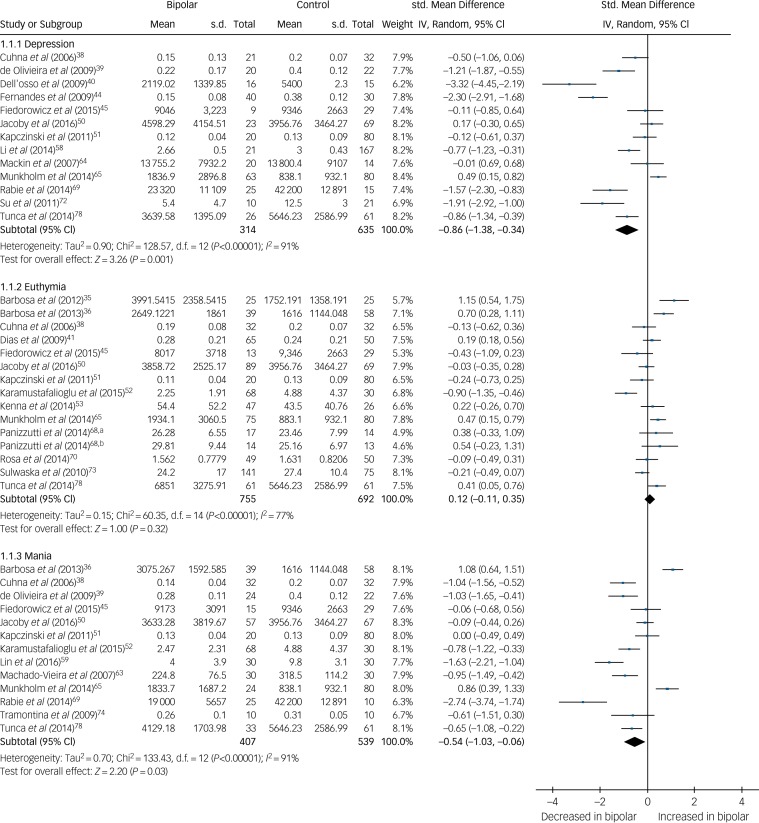
Forest plot of brain derived neurotrophic factor (BDNF) in depression, euthymia and mania compared with healthy controls. a. Early stage; b. Late stage. Std, standard.

There were 30 studies investigating neurotrophins. BDNF levels were significantly decreased in both mania (SMD −0.54, 95% CI −1.03 to −0.06, *P* = 0.03, *I*^2^ = 91%, *k* = 13, *n* = 946) and bipolar depression (SMD −0.86, 95% CI −1.38 to −0.34, *P* < 0.00001, *I*^2^ = 91%, *k* = 13, *n* = 949), whereas in euthymia there was no significant difference (SMD 0.12, 95% CI −0.11 to 0.35, *P* = 0.32, *I*^2^ = 77%, *k* = 14, *n* = 1447) (Fig. 4). A further three studies investigated BDNF in euthymia finding decreased levels in some subgroups,^86^^,^^88^^,^^90^ but did not provide data to allow inclusion in the meta-analysis, with findings shown in supplementary Table 1.

Meta-analysis of three studies showed neurotrophin-3 levels were not significantly different from healthy controls in bipolar depression (supplementary Fig. 9). There were insufficient studies to conduct a meta-analysis for the other mood phases.

### Oxidative stress markers

Ten studies were available, of which, two investigated TBARS in each mood phase. In both, TBARS was increased in mania and bipolar depression. One found TBARS to be elevated and another decreased in euthymia (supplementary Fig. 10). There were too few studies to conduct meaningful meta-analyses of other oxidative stress markers.

### Medication-free subgroup

A subgroup analysis was performed for the 15 studies that investigated biomarkers in patients that were free from all psychotropic medication at the time of assessment, and forest plots are presented in supplementary Figs 11–14. The results in medication-free patients were not found to change significantly from the overall results in each meta-analysis. Meta-analyses with three or more studies were performed for BDNF, IL-4, TNF-α and IFN-γ in patients with mania and BDNF for bipolar depression. There were insufficient studies to complete meta-analyses for any biomarker in euthymia in patients who were medication free.

BDNF was found to be decreased in both mania (SMD −1.16, 95% CI −1.5 to −0.81, *P* < 0.00001, *I*^2^ = 32%, *k* = 4, *n* = 252) and bipolar depression (SMD −1.35, 95% CI −2.11 to −0.6, *P* = 0.0004, *I*^2^ = 69%, *k* = 3, *n* = 251). TNF-α was increased in mania (SMD 3.69, 95% CI 1.28 to 6.09, *P* = 0.003, *I*^2^ = 96%, *k* = 3, *n* = 212). IL−4 and IFN-γ were not found to be significantly different when participants with mania where compared with healthy controls.

### Discriminatory biomarkers

There were a sufficient number of studies to analyse five biomarkers across each mood phase of bipolar disorder. sTNFR1 is raised in mania but is not significantly different in bipolar depression or euthymia. BDNF is significantly decreased in both mania and bipolar depression, whereas TNF-α is raised in mania and bipolar depression, and neither are significantly different from levels in controls in euthymia. hsCRP and IL-6 are raised in both euthymia and mania but are not significantly different from controls in bipolar depression. A combination of these biomarkers appears to be differentially altered in each mood phase, as described in Table 2. A summary of the effect sizes of these biomarkers in each mood phase is shown in supplementary Fig. 1.

**Table 2:** Fully discriminatory biomarker combinations

Bipolar mood phase	hsCRP/IL-6	sTNFR1	BDNF	TNF-α
Euthymia	↑	↔	↔	↔
Depression	↔	↔	↓	↑
Mania	↑	↑	↓	↑

hsCRP, high-sensitivity C-reactive protein; IL, interleukin; sTNFR1, soluble tumour necrosis factor (TNF)-α receptor 1; BDNF, brain derived neurotrophic factor; ↑, higher in case participants; ↔ , not significantly different from controls; ↓, lower in case participants.

In addition, we present three further biomarkers (IL-1RA, sIL2-R, sIL6-R) (described in Table 1) that require further work in specific mood phases that did not have sufficient studies to be included in a meta-analysis.

## Discussion

This systematic review and series of exploratory meta-analyses report results that include neurotrophic, inflammatory and oxidative stress biomarkers, separated by affective state (euthymia, mania, depression) in bipolar disorder. We were able to include 53 studies comprising 2467 participants and 2360 healthy controls, synthesising data on 14 different biomarkers. A combination of hsCRP/IL-6, BDNF/TNF-α and sTNFR1 appear to be differentially altered in each mood phase.

Previous meta-analyses have investigated specific biomarkers in bipolar disorder, including hsCRP, cytokines, BDNF and oxidative stress markers. However, this review is the first to combine multiple circulating blood-derived biomarkers separated by mood phase in bipolar disorder. Important results can be unmasked in this type of finer grain analysis that have the potential to bring translational impact a step closer. This review may help to shape future research strategies to translate biological findings into clinical practice. Increased awareness of the pathophysiological and biochemical differences that separate not only the varied poles present in bipolar disorder, but also those that differentiate bipolar disorder from other affective illnesses such as unipolar depression, may help to facilitate expedited diagnosis and hint towards future pharmacological targets for bipolar disorder.

### Main findings: potential discriminatory and non-discriminatory biomarkers

We present a combination of five biomarkers that appear discriminatory based upon our proposed definition. These are hsCRP/IL-6, BDNF/TNF-α and sTNFR1 shown in Table 2. sTNFR1 appears to discriminate mania only, where it is significantly different from controls. Levels of BDNF, hsCRP, IL-6 and TNF-α are able to discriminate either euthymia or depression by being no different in that mood phase in comparison with levels in controls but altered in other mood phases. Both hsCRP and IL-6 discriminate bipolar depression, their levels being no different from controls, whereas BDNF and TNF-α are significantly altered in both mania and depression and not significantly different in euthymia compared with controls, and therefore may be more generalised markers of affective disturbance in bipolar disorder. In our analysis, there were no biomarkers that would individually be able to discriminate each mood phase. However, our findings suggest the combination of hsCRP/IL-6, sTNFR1 with BDNF/TNF-α would meet this criterion.

We also describe three biomarkers that may have the potential to be discriminatory, although further research is required. For example, IL-1RA was raised in mania but not significantly different from lvels in controls in euthymia. The two studies investigating IL-1RA in bipolar depression also found levels not significantly different from controls, and if confirmed in further studies this would support the potential of this marker to discriminate bipolar mania. sIL-2R and sIL-6R may have the potential to be discriminatory although only when combined with other biomarkers, and future research is required of these markers in the bipolar depression phase.

Levels of IL-2, IL-4, IL-10 and IFN-γ were not significantly different from controls in either mania or euthymia, but current study numbers mean no conclusions can be reached regarding levels in bipolar depression; further studies are required. Assessing only patients free from psychotropic medications at the time of biomarker measurement did not affect the significance or direction of the main results, supporting the direction and validity of the main results.

### Comparisons with other research

#### BDNF

This meta-analysis found decreased BDNF levels in both mania and depression, but not euthymia. A particularly large effect size was seen in depression (SMD −0.86) and a moderate effect size in mania (SMD −0.54). In fact, BDNF was the only biomarker in our analysis to be significantly decreased in comparison with healthy controls, in any mood state. These findings are in line with other research into bipolar disorder,^7^^,^^12^^,^^14^ but it is also noteworthy that decreased BDNF is associated with schizophrenia and unipolar depression.^96^^,^^97^

#### IL-6, sIL-6R and hsCRP

IL-6, sIL-6R and hsCRP were found to be increased in euthymia and mania, but not in bipolar depression (for sIL-6R insufficient studies were available to conduct a meta-analysis for depression). This may add to the validity of our results as there is evidence linking the inflammatory pathways of these markers; IL-6 is one of the primary cytokines in the inflammatory cascade, which stimulates production of CRP,^98^ and its proinflammatory action is mediated by its soluble receptor sIL-6R.^99^

There is relatively strong evidence that hsCRP and IL-6 are raised in unipolar depression,^100^^,^^101^ whereas our results suggest this is not the case in bipolar depression, although it is unclear whether this may be because of methodological differences between studies. Nevertheless, this not only raises the possibility of a useful biomarker to differentiate bipolar disorder from unipolar depression, but points to the differing pathophysiology of the disorders and differences in their respective inflammatory profiles.

#### TNF-α, sTNFR1 and sTNFR2

TNF-α has been described as one of the primary inflammatory mediators,^102^ driving production of other cytokines such as IL-6 and CRP,^98^ all of which were found to be elevated in bipolar mania in our analysis. TNF-α and its soluble receptors have been implicated in neurocognition in bipolar disorder,^103^ and previous meta-analyses have found TNF-α and sTNFR1 elevated in mania^6^ but it remains unclear what role sTNFR1 and sTNFR2 have during the depressive mood phase.

### Oxidative stress markers

Oxidative stress markers have been investigated in previous meta-analyses,^18^^,^^19^ although these did not separate results by affective state. Oxidative stress markers have been implicated in multiple psychiatric disorders including schizophrenia,^104^ depression, anxiety, dementia and substance misuse.^105^ TBARS in particular appear to be increased in schizophrenia,^105^ although not in unipolar depression,^106^^,^^107^ indicating a possible differential biomarker from bipolar depression if results are confirmed by further studies.

### Within-participant differences

Several studies examined changes in biomarkers levels within participants following treatment or a change in mood phase. Findings were generally inconsistent for the biomarkers under investigation; for example, studies showing BDNF levels may increase,^74^ decrease^52^ or remain unchanged.^50^^,^^59^^,^^65^ between manic episode and subsequent remission. Some studies went beyond assessment of difference in biomarker level between mood phases, finding that decreases in sIL-2R levels between mania and subsequent remission correlated with an improvement in symptoms.^75^^,^^77^ Individual changes in biomarker levels between mood phases and how this correlates with treatment or clinical improvement is currently understudied.

### Interaction of biomarkers

The markers identified in this review are unlikely to be altered in isolation. There is evidence that multiple pathways interact to cause downstream effects. Neuroinflammation appears to cause dysfunction in several neurotransmitter systems and neuronal signalling, whereas proinflammatory cytokines such as TNF-α and Il-6 that this review found to be raised in bipolar mania activate microglia that release reactive oxygen species contributing to oxidative damage, protein aggregation and apoptosis.^102^ These changes in glial function then decrease production of neurotrophic factors such as BDNF.^108^ These interactive effects may be one reason that a biomarker panel approach may be worthwhile.

### Strengths and limitations

This comprehensive systematic review includes a series of exploratory meta-analyses of inflammatory, neurotrophic and oxidative stress markers in bipolar disorder. This review separated the results of each biomarker by mood phase, which not only provides information as to physiological changes, but also reduces the risk of type 2 error where there are true state-related changes in biomarkers. The use of random-effects models in each analysis also adds further validity to the results.

Notwithstanding this, there are several limitations and results should be interpreted with some caution, in part because of the nature of the literature reviewed. The robustness of our results is dependent on the quality of the primary studies, most of which were observational and at significant risk of bias and confounding. The significant methodological heterogeneity between included studies becomes problematic when attempting to use biomarkers as a diagnostic tool in bipolar disorder and means our results and conclusions should be considered tentative. The individual patient characteristics varied greatly between studies, and factors such as body mass index, blood pressure, physical activity and smoking status, which were heterogeneously addressed among studies, can affect biomarker levels.^109^^–^^111^ Factors such as duration of the illness or mood phase, the specific assay used and whether the biomarker was measured in plasma or serum may also influence biomarker levels.^7^^,^^112^ The inclusion of patients with a diagnosis of bipolar disorder type II or hypomania, and variation in the YMRS and HRSD scores used to define mood phases are likely to have contributed to the significant heterogeneity in the results of the meta-analyses and introduced potential confounders. In addition, the definition of ‘healthy controls’ varied considerably. Furthermore, as studies that investigated multiple biomarkers compared these with a single control group, these are repeated in the separate meta-analyses, and any abnormalities in these control groups may be amplified.

Most studies also included both patients taking medicated and those not, and many psychotropics such as lithium, valproate and antipsychotics are known to affect biomarker levels.^91^^–^^94^ Several studies also reported biomarker levels following successful treatment and remission, and it is unclear if there is a lag time to normalisation of the biomarker. However, including only patients free from psychotropic medications at the time of assessment did not appear to change our main findings.

The biomarkers investigated in this review, particularly pro- and anti-inflammatory cytokines^113^ may be correlated and we could not account for this in our analysis. Future studies moving towards translation to a clinical test need to account for this.

Mixed states have long been known to occur in bipolar disorder but undoubtedly present a nosological problem^114^^,^^115^ and as such a major challenge in linking the clinical picture to biomarker levels. We cannot be clear to what extent this phenomenon will have had an impact on the primary studies as well as our results. Although some studies did investigate biomarkers in those with a diagnosis of mixed affective state these were not of sufficient number to conduct meaningful analysis. However, it is likely that especially in older studies, some people diagnosed as having mania or depression may have had a mixed states picture.

The issue of publication bias has been raised in relation to biomarkers in bipolar disorder, with concern that positive results are more likely to be published.^116^ However, in this meta-analysis examination of funnel plots with sufficient number of studies resulted in no obvious asymmetry, and a number of negative results were identified, leading to several findings of biomarkers that appear to be unchanged in bipolar disorder. It is therefore unlikely that an excess of positive results affected the overall results of the meta-analysis. However, as many analyses included too few studies for funnel plots to be meaningfully interpreted, it is unclear if publication bias may be a factor in some of these less extensively investigated markers.

Because of the comprehensive scope of our review and the need to separate results by mood phase, we conducted a considerable number of meta-analyses, and this increases the risk of type 1 error. Applying a correction for multiple testing is likely to exclude biomarkers that are promising but for which there are too few studies. As the purpose of the review was exploratory as opposed to hypothesis testing, we did not correct for multiple testing, and enable readers to understand the full breadth of the extant literature. However, it is important to consider this and therefore exercise caution in weighing and using our findings. Equally, because of the exploratory nature of the review we were unable to prospectively perform a sample size calculation for each biomarker, and therefore it is likely that for some biomarkers the meta-analyses are underpowered as a result of the small number of studies included.

### Implications and future directions

The identification of discriminant biomarkers has potentially significant clinical relevance through increasing diagnostic accuracy, monitoring of disease severity, predicting changes in mood phase as well as providing information about the underlying neurobiology of bipolar disorder. Although there remains inconclusive evidence at present, a number of biomarkers show promise in their ability to differentiate mood phases in people with bipolar disorder from healthy controls, and potentially from other mental illnesses that may present similarly to bipolar disorder and thus make clinical diagnosis challenging. This review investigated state-related biomarkers. However, markers such as BDNF and TNF-α are associated with illness duration,^86^ whereas alterations in IL-6 during childhood are associated with onset of hypomanic symptoms in adulthood.^117^ Therefore, further investigation is required of how far the identified biomarkers represent longer term markers of illness, as well as markers of mood phase. Prospective cohort studies with repeated measures of these biomarkers are necessary to determine the temporality of biomarker change with mood phase, exclude confounders such as illness duration and medications, and determine the predictive value of such biomarkers.

Finally, the comparisons of our findings with other literature have identified some potential biomarker differences between specific mood phases in bipolar disorder from other major mental illnesses that can present similarly, such as IL-6 in and hsCRP in unipolar and bipolar depression. Further longitudinal research in this area may help to unearth strong candidates for biomarkers that can differentiate bipolar disorder from other psychiatric disorders that have biomarker associations such as unipolar depression and schizophrenia.

In conclusion, this meta-analysis has identified neurotrophic, inflammatory and oxidative stress markers that are altered during different mood phases of bipolar disorder. Most significantly, a combination of hsCRP/IL-6, BDNF/TNF-α and sTNFR1 may offer the potential to differentiate people with bipolar disorder from matched controls specific to bipolar mood phase.

## Supplementary material

For supplementary material accompanying this paper visit http://doi.org/10.1192/bjp.2018.144. click here to view supplementary material
