# Supplementary material for: Neurotrophins, cytokines, oxidative stress mediators and mood state in bipolar disorder: systematic review and meta-analyses
Source: Br J Psychiatry. 2018 Sep;213(3):514–25. doi: 10.1192/bjp.2018.144 (PMC6429261; doi:10.1192/bjp.2018.144)
Supplement: Supplementary file 1 [file S0007125018001447sup001.zip › S0007125018001447sup001/Supplementary data Figure 1 - Differential biomarker levels in depression, euthymia and mania.pdf]

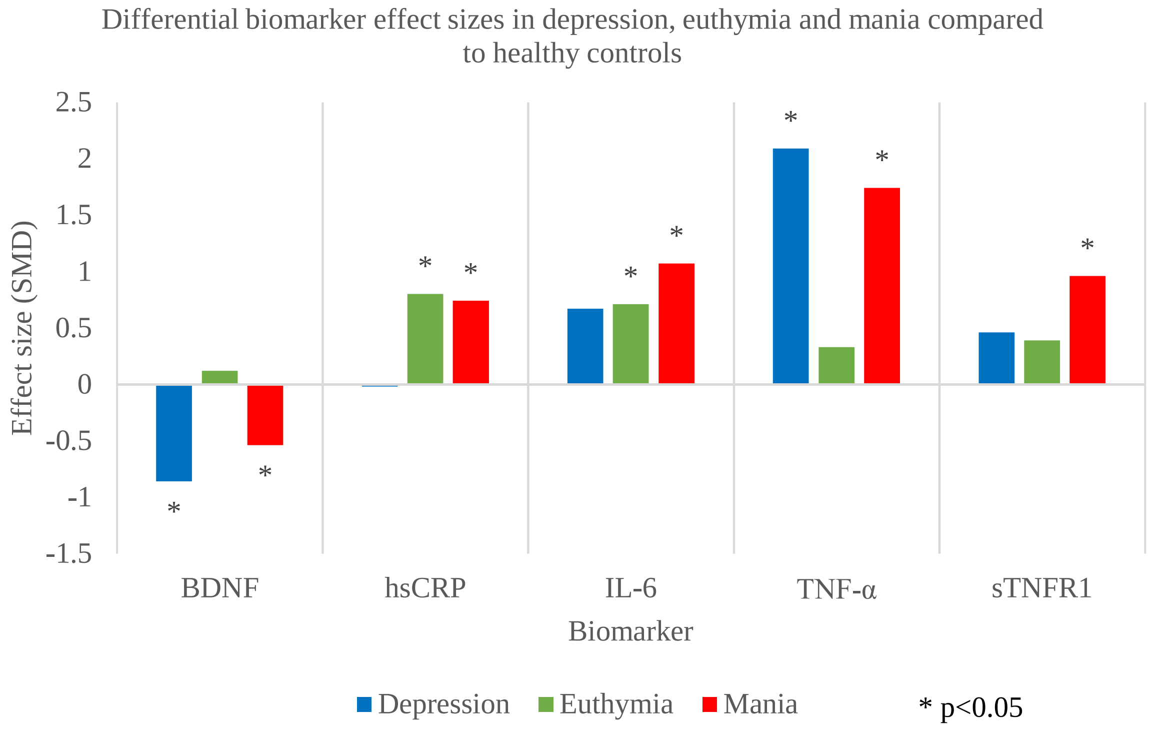
Differential biomarker effect sizes in depression, euthymia and mania compared to healthy controls
Effect size (SMD)
2.5
2
1.5
1
0.5
0
-0.5
-1
-1.5
*
*
*
*
*
*
*
*
*
BDNF
hsCRP
IL-6
TNF-α
sTNFR1
Biomarker
Depression
Euthymia
Mania
* p<0.05
