# Supplementary material for: Neurotrophins, cytokines, oxidative stress mediators and mood state in bipolar disorder: systematic review and meta-analyses
Source: Br J Psychiatry. 2018 Sep;213(3):514–25. doi: 10.1192/bjp.2018.144 (PMC6429261; doi:10.1192/bjp.2018.144)
Supplement: Supplementary file 1 [file S0007125018001447sup001.zip › S0007125018001447sup001/Supplementary data Table 1 - Characteristics of Included Studies table.docx]

Supplementary Table 1: Characteristics of Included Studies

| **Author & Year (country)** | ***N cases, controls***  ***Age mean (SD), sex*** | **Biomarkers measured** | **Inclusion criteria** | **Exclusion criteria** | **Assay/Test type** | **Meds** | **Summary of findings** |
| --- | --- | --- | --- | --- | --- | --- | --- |
| Inflammatory Markers | | | | | | | |
| Bai et al (2014)  (32) (Taiwan) | *130,130 46.1(9.3) F 78.6%* | CRP, sIL-2R, sIL-6R, sTNF-R1, sP-selectin, MCP-1 | Cases: DSM IV BD. MADRS (>12 depression) & YMRS (<9 euthymia >9 mania) Controls: - | Both: Comorbid DSM dx; substance use; pregnancy; breastfeeding. | ELISA | MS, AP | CRP, IL2R, IL6, IL2R, TNFαR, MCP-1 *p=<0.0001* P-selectin *p=0.11* |
| Barbosa et al (2011) (33) (Brazil) | *34, 38 42.9(9.7) F 52.6%* | TNFα, sTNFR1, TNFR2 | Cases: BD type 1. HDRS, YMRS.  Controls: healthy, local population, no personal or family history of psychiatric disorder | Infection <1month, physical meds, dementia Controls: + no family psychiatric history | ELISA | Li, MS, AP, AD | TNFR1 *p<0.001* TNFR2 p=0.48 |
| Barbosa et al (2012) (35) Brazil | *25, 25 50.8(9.1) F 52%* | BDNF, TNFα, sTNFR1, STNFR2 | Cases: M.I.N.I. Plus to confirm BD type 1 (DSM IV), >18yrs, YMRS & HRDS <7 euthymia Controls: Matched by gender, age, education | Both: Physical illness, organic disorder, other medication <4 weeks | ELISA | Li, AP, MS | increased BDNF (p=0.001) in cases |
| Breunis et al (2003) (83) (US, Holland) | *64,24* | NK, B, T  TPO-Ab, sIL2-R | Cases: BD (SCID), inpatients. DSM IV, type 1 & 2 Controls: - | - | ELISA | - | NK, B, T lymphocytes all p>0.05 IL2-R p<0.001 |
| Brietzke et al (2009) (84) (Brazil) | *61,25 43.4(12.3) F 80.0%* | TNFα, IL2, IL4, IL6, IL10, IFNγ | Cases: BD type 1 diagnosis by SCID-I divided into manic, depressive, euthymic groups with YMRS, HRDS (<8 euthymia, >7 YMRS mania, >7 HDRS depression) used. Control: healthy by SCID-I | Cases: No medical history, smoke <10/day, meds Controls: no medications, no psychiatric history, dementia, cancer, tumour, no family psychiatric history | CBA | - | IL2,4,6 p<0.05 IL10, IFNγ, TNFα P>0.05 euthymia>control P<0.05, depressed>control p<0.05 manic>control p<0.05 |
| Cetin et al (2012) (37) Turkey | *45,23 31.2(5.2) F 52.2%* | TNFR-1, IL-6R, IL-2R | Cases: DSM IV diagnosis BD type 1 by consensus decision of SKIP-TURK Controls: “healthy” | Both: Allergic disease, infectious disease <4weeks, immunosuppressants, pregnancy, breastfeeding, alcohol >5units, >3 coffees, >10 cigarettes | ELISA | MS, AP | TNFR1, sIL6R p<0.05  manic>euthymic p>0.05 |
| De Berardis et al (2008) (85) Italy | *30, 30, 30 34.5 (8.8) F 54%* | CRP, TC | Cases: BD-type 1 (DSM-IV), SCID-I, 18-65 years. YMRS>19 Controls: Healthy volunteers (local) with no psychiatric or medical illness | Both: Axis-1 comorbidity, organic disorder, substance use, pregnancy, no medical history, obesity | ELISA | None | TC levels were lower in all clinical groups compared to controls. TC levels were negatively correlated to BRMRS, YMRS and HAM-D. |
| do Prado et al (2013) (42) Brazil | ***27,24 45.7(9.2) F 100%*** | IL2, IL4, IL6, IL10, IL17, IFNγ, TNFα, MAPK | Cases: Outpatients, SCID-I BD type 1. HDRS & YMRS (euthymia <8).  Controls: Age and sex matched | Both: Presence of axis I disorder, history of severe medical history, brain injury, systemic disease, inflammatory states, substance use | CS | Li, AD, AP, MS | BD patients higher IL-2, IL-4, IL-5, IL-10, IL-17, IFN-γ and TNF-α compared to controls (p<0.05) |
| Drexhage et al (2011) (43) Holland | 38,22 41.1(9.6) F 80% | PTX3, CCL2, TNFα, IL1β, IL6, IL8, IL10; IFNγ, IL4, IL5, IL17A, IL22 | Cases: 18-60, BD type 1 or 2 by DSM IV, twin pairs, by SCID. YMRS (<4) & IDS (<12) (all euthymic) Controls: - | Cases: No alcohol, drug use <6months, no ‘severe’ medical illness. Controls: no DSM diagnosis (SCID), no family psychiatric history | CBA | - | All insignificant, though noted many at lower end / below detection level of assay |
| Fiedorowicz et al (2015) (45)  USA | *37, 29*  *41(13)*  *F 35%* | TNFα, sTNFR1, sTNFR2, IL1β, IL6, IL8, IL10, IL4, IFNγ, MCP1, VEGF | DSM IV BD. Mania = YMRS >20, depression = MADRS >20, euthymia YMRS <12 & MADRS <10. Inpatients and outpatients. Controls: matched for age and sex. Recruited through local adverts | Drug, alcohol use, psychiatric comorbidity, physical illness Controls: No history psychiatric or physical illness | ELISA | Li, MS, AD, AP | BD & controls no significant differences.  mania & depression elevated TNF-α, sTNFR1/sTNFR2, IL-1β,  IL-6, IL-10, IL-18, IL-4, interferon-γ compared to euthymia |
| Guloksuz et al (2010) (47)  Turkey | *31, 16*  *31.8(4.8)*  *F 25%* | IFNγ, TNFα, IL2, IL4, IL5, IL10 | Cases: DSM IV BD, outpatients, YMRS (>7), HAM-D, SCID-I. Controls: Matched for age, gender, ethnicity; from hospital staff | No recent inflammatory illness, no immunosuppressants, NSAIDS, steroids, alcohol dependence, pregnancy, heavy smoking/caffeine use, Controls: No regular medication, no axis 1 disorder, no family psychiatric history. | CBA | None / Li | No differences in cytokine (euthymic-BD and controls). TNF-α and IL-4 euthymic-BD patients higher than in both the euthymic-BD patients and controls. |
| Huang and Lin (2007) (49) Taiwan | *13,23,31*  *35.7(10.1)* | hsCRP | DSM IV SCID, inpatients BD type 1 (YMRS>26), outpatient depression, HDRS (>20) Controls: - | Medical co-morbidity, no medication <1 week Controls: - | PEI | None | BD > controls p=0.043 depression > controls p=0.172 |
| Hope et al (2011) (48) Norway | *112,153,*  *239 36.2(12) F 60%* | sTNF-R1, IL1-Ra. hsCRP, OPG, vWF | Cases: 18-65yrs, inpatient/outpatient, DSM IV BD, SCID, GAF, IDS (<14 euthymia >14 depression), YMRS (<8 euthymia >7 mania), CDSS Controls: - | Cases: head injury, neurological disorder. Anti-inflammatories, acute illness Controls: no past medical history/DSM diagnosis | EIA | AP, Li, AD | BD elevated>depressed sTNF-R1, IL-1Ra, OPG, IL-6 p<0.05; vWF hsCRP p>0.05 BD neutral > control OPG, IL-6 p>0.05 BD elevated > control sTNF-R1, vWF p>0.05 Total IDS –ve correlated OPG, IL-1Ra, IL-6 p<0.05 |
| Jacoby et al (2016) (50)  Denmark*┼ | *60, 35*  *42.7(11.7)*  *F 38.3%* | BDNF, hsCRP, IL1β, IL6, IL8, IL18, TNFα | Cases: ICD-10 BD type 1, 18-60, outpatients, SCAN interview.  Controls: blood donors at blood bank, 18-60. | Cases: Significant medical disorder, substance use, pregnancy, use of restraint. Controls: no psychiatric illness, family history, significant medical disorder, substance use, pregnancy. | ELISA | Li, MS, AP | IL-6 & IL-8 were increased in BD compared to controls (p<0.05) |
| Kauer-Sant'Anna et al (2009) (86)  Canada┼ | *60, 60*  *36.4(8.4)*  *F 54%* | BDNF, TNFα, IL6, IL10 | Cases: DSM IV BD type 1, inpatients (first episode, 15-35) and outpatients (late stage, 18-65), SCID-I and MINI, YMRS, HDRS. Controls: matched for age, gender, education. | Cases: Comorbid medical conditions, other medications Controls: no psychiatric or physical illness, recent infection, non-smokers. | ELISA | MS, AP, AD | BDNF was decreased in late-stage-BD. BDNF negatively correlated with length of illness. All interleukins and TNFα increased in early stages of BD, compared to controls. |
| Kim et al (2007) (54) S Korea | *37,74 37.8(12.0) F 62%* | TNFα, IL-6, IL-4, IFNγ, IL-2 | Cases: DSM IV SCID BD manic, normal FBC, U&E, VDRL, ECG, EEG. Controls: Normal FBC, U&E, TFT, LFT, CXR, ECG, EEG. | No medication <4 months, no infective illness, autoimmune disease, substance use.  Control: no personal or family psychiatric history. No medication. | ELISA | None | IL-6 & TNFα cases>controls p<0.0001 IL-4 cases<controls p<0.0001 IL-2 IFNγ p>0.05 |
| Kim et al (2004) (55) S Korea*┼ | *70, 96 32.9(12.2) F 67%* | IFNγ, IL-4, TGF-β1 | Cases: DSM IV SCID, all ‘manic or active symptoms’. BPRS, YMRS. Controls: local screening. Normal bloods, ECG, EEG. | Cases: No infective illness, autoimmune disease, substance use.  Control: no personal or family psychiatric history. No medication, no substance use, free of ‘chronic and acute physical illness’. | ELISA | None | IFNγ, IL-4 cases>controls p<0.0001 TGF-β1 cases<controls p=0.001 After 8 weeks treatment IFNγ, IL-4 no significant difference TGF-β1 increased p=0.002 |
| Kim et al (2002) (56) S Korea | *25,34,43,85 34.6(11.4) F 65%* | IL-12 | Cases: DSM IV SCID, all ‘acute’. BPRS, HAM-D. Normal bloods, ECG, EEG. Controls: local screening. Normal bloods, ECG, EEG . | Cases: No psychiatric comorbidity. Controls: no psychiatric history or family psychiatric history, autoimmune/infectious disease, substance use | ELISA | None | No significant difference BD/controls at baseline IL12 significantly lower at 8 weeks p=0.04 |
| Kunz et al (2011) (87) Brazil | *20,53,80* | IL-6, IL-10, TNFα | Cases: DSM IV SCID. BPRS (<25), CGI, YMRS. Controls: - | Cases: Non-smokers, non-obese, no ‘major’ medical condition. Controls: no personal or family psychiatric history. | ELISA | AP | IL-6, TNFα no significant difference controls/BD IL-10 controls<BD p=0.004 |
| Legros et al (1985) (57)  Belgium | *92, 150 42(?)* | IgM, IgG, IgA | Cases: BD diagnosis by criteria of Feighner et al (1972). BD depression. Control: - | Cases: Substance use, organic psychosis, somatic illness. Control: - | CSRI | None | IgM significantly increased BD > controls. |
| Liu et al (2004) (60) Taiwan | *29,20 32.8(8.9)* | IL-1RA, sCD4, sCD8, IFNγ, IL-2, IL-4, IL-10 | Cases: DSM-IIIR BD mania YMRS >26. Age 16-44, physically healthy. Controls: age, sex matched. ‘healthy’ from hospital staff & students. | Cases: Comorbid substance use Controls: no infection, allergies, immune disease. No use of drugs ‘that alter endocrine function’ | ELISA | Li, AP, MS | IL-1RA, sCD4, SCD8, IFNγ cases > controls p<0.05  IL-2, IL-4, IL-10 not significant |
| Lotrich et al (2014) (62)  USA | *21, 26*  *65.5(8.4) F 53.9%* | IL1RA | Cases: DSM IV SCID BD 1-2, euthymia, Inpatients and outpatients, age >50, HDRS <10. Control: matched for age, education, gender, cardiovascular burden. Recruited through health fayres and adverts. | Organic brain disorder, ECT <6 months, substance use <12 months.  Control: no physical or psychiatric comorbidity. | - | - | IL-1RA was elevated in BD subjects compared to controls (439+/−326 pg/mL vs. 269+/−109 pg/mL; p=0.004). |
| O’Brien et al (2006) (66) Ireland | *21,21 41.17(?) F 64.2* | IL-6, IL-8, IL-10, TNFα, sIL-6R | Cases: DSM IV BD, ‘physically healthy’, depression (HRDS >17), mania (YMRS >20). Controls: - | Cases: <2 weeks: allergic, infectious, inflammatory diagnoses. Axis 1 psychiatric history. Control: No personal or family history of mood disorder. | ELISA | Li,MS,AP | Cases > controls IL-8 (p<0.001), TNFα (p<0.05)  No significant difference IL-6, IL-10, sIL=6R |
| Ortiz-Dominguez et al (2007) (67) Mexico | *20,33 32.3(10.8) F 87%* | TNFα, IL-6, IL-1B, IL-2, IL-4 | Cases: BD type 1 20-50yrs, >9 years education, middle socio-economic class. DSM IV SCID, inpatient & outpatient, YMRS >20. Controls: matched age, gender, ethnicity and demographic> | Cases: Infectious disease, allergies, medical illness, no comorbid axis 1 illness. Controls: no psychiatric disorder, free of medication 3 weeks physically healthy. | ELISA | None | Cases>controls TNFα p<0.05  Cases <controls IL-1β p<0.05. depression & mania not significant  Cases>controls p<0.05. depression & mania not significant  Cases >controls IL-4. Mania > depression p<0.05  Cases<controls p<0.05 IL-2. Depression & mania not significant |
| Panizzutti et al (2015) (68) Brazil┼ | *31, 27*  *40.7(14) F 86%* | BDNF, TBARS, GPx, IL2, IL4, IL6, IL10, IL17, TNFα, IFNγ, CCL11, CCL24 | DSM IV SCID -I, BD type 1, early and late stage, YMRS, HDRS  Control: matched for sex and age. | Cases: History of autoimmune disease, chronic infections, immunosuppressive therapy Controls: autoimmune disease, immunosuppressive therapy, family or personal history of psychiatric disorder. | ELISA | Li, MS, AP, AD | eotaxin/CCL11 higher in late-stage patients with BD compared to controls. |
| Su et al (2011) (72)  Taiwan┼ | *10, 21 22.2(?) F 0%* | BDNF, CRP, TNFα, IL-6 | Cases: Inpatients, BMI <25, no smoking <1 week. Diagnosis by DSM IV criteria. BD depression. Control: matched for age & BMI. From hospital staff, HAM-D <7. | Cases: Diabetes, family history of diabetes, renal disease, thyroid disease, liver disease, chronic inflammatory disease. Control: no history hypomania, no medication. | ELISA | None | BDNF lower in controls. No significant differences in other markers |
| Tsai et al (1999) (75) Taiwan | *23,23 30.6(8.9) F 54%* | PHA, concanavalin A, sIL-2R, sIL6-R, | Cases: inpatients, BD mania, DSM IIIR, PDA, YMRS>26 mania <12 remission, <45yrs, “physically healthy”. Controls: from hospital staff & local church youth group, normal bloods. | Cases: Current mixed episode or substance use disorder, past history of inflammatory disorder, infection <2 weeks. Control: Axis 1 psychiatric disorder, ‘physically healthy’. | ELISA | Li, AP | sIL2-R higher in remission than mania (p<0.025) and controls (p<0.001)  No significant difference sIL6-R: mania=remission=controls  PHA mania>remission (p<0.025), no significant difference controls |
| Tsai et al (2001) (77) Taiwan | *31,31 33.1(8.7) F 54%* | sIL2-R, sIL-6R | Cases: DSM IV BD mania, PDA, YMRS >26 (mania) <12 (remission), normal physical exam, bloods. Controls: Age and gender matched, hospital staff and students and local church youth group. Screened with GHQ (Chinese) for ‘psychiatric disturbance’. | Cases: Mixed episode, other axis 1 disorders, autoimmune/recent infection <2 weeks. Controls: recent infection <2 weeks, autoimmune history, GHQ >4. | ELISA | Li, AP | sIL2-R mania<remission (p<0.05) and higher than controls (p<0.0005)  sIL6-R no significant difference mania, remission or control. No p values given |
| Tsai et al (2012) (76) Taiwan | *33, 33 23.2(7.0) F 33%* | Hs-CRP, IL1-Ra | Cases: DSM IV BD I <45, acute inpatients, YMRS >26 (mania) <5 (remission) HAMD <7 (remission) for 8 weeks, PDA. Controls: age and gender matched. | Both: Other axis 1 disorder (GHQ), history of autoimmune disease, endocrine illness, infection, allergies, medication that could affect cytokine, lipid, endocrine levels. | ELISA | Li, AP | IL1-RA mania & partial remission > controls (p<0.05)  hsCRP mania & remission > controls (p<0.05).  No significant correlation IL1-RA & hsCRP.  hsCRP full remission > partial remission (p<0.05) but not higher than mania (p=0.63). |
| Uyanik et al (2015) (79)  Turkey | *30, 28*  *33.4(8.6)*  *F 46%* | TNFα, IFNγ, IL6, hsCRP | Cases: DSM IV BD I, 18-65yrs. Controls: - | Cases: No other psychiatric history, pregnancy, breast feeding, autoimmune disorders, infection <2 weeks, substance/alcohol/caffeine heavy use, organic disorder. Controls: chronic disease, using NSAID, antidepressants <1 month, BMI >30, smoker | ELISA | None | TNF-α, INF-γ, IL-6 and hsCRP  Levels were significantly higher in patients with manic episode of BD I disorder before treatment than HS. After treatment the levels of TNF-α, INF-γ, IL-6 and hsCRP were observed to be significantly decreased |
| Wadee et al (2002) (81)  South Africa | *45,45*  *32.7(?)*  *F 40%* | CRP, CIC, immunoglobulins, complement | Cases: DSM IV MINI interview, BD (mania), 18-65 years, inpatients. Controls: matched for age. | Both: Abnormal LFTs, TFTs, acute medical disorder, no recent use of other medications, no substance/alcohol use, pregnancy | PEN | - | immunoglobulin D cases<controls, total immunoglobulin and immunoglobulin G1, complement proteins C3, C6 and Factor B cases >controls. |
| Neurotrophins | | | | | | | |
| Barbosa et al (2013) (36) (Brazil) | *87, 58 49.6(?) F 67.8%* | BDNF | Cases: BD type 1, MINI plus  HDRS, YMRS. Remission: <7 for 8 weeks. Controls: healthy, local population, no personal or family history psychiatric disorder. | Cases: Dementia, infectious, autoimmune disease, anti-inflammatories, steroids, antibiotics <1 week. Controls: no psychiatric disorder/family history psychiatric disorder. | ELISA | Li, MS, AD, AP | BDNF (p<0.001) BDNF mania > remission (p=0.34) |
| Barbosa et al (2014) (34) (Brazil) | *40, 18*  *49.7(?)*  *F 72.5%* | NT-3, NT 4/5 | Cases: BD type 1, MINI plus  HDRS, YMRS. Remission: <7 for 8 weeks Controls: healthy, local population, no personal or family history of psychiatric disorder | Both: no previous psychiatric disorder or family history of psychiatric disorder, cognitive deficits | ELISA | Li, MS, AD, AP | NT3: BD (manic)=BD (remission)=controls (p>0.05)  NT4/5: BD (manic)<controls (p<0.05)  BD (manic)=BD (remission) (p>0.05)  BD (remission)= controls (p>0.05) |
| Cunha et al (2006) (38) Brazil | *74,32 40.7(12.1) F 65.6%* | BDNF | Cases: DSM IV diagnosis BD type 1, SCID. YMRS, HDRS (<7 euthymia).  Controls: matched. | Both: Smoking, no medication, no comorbidity, no family history psychiatric disorder. | ELISA | Li, MS, AD, AP | BDNF lower in manic than in depressed and euthymic (p<0.05) |
| de Oliveira et al (2009) (39)  Brazil | *22,22 39.8(12.9) F 66%* | BDNF | Cases: BD type 1 (DSM IV) inpatients using SCID I, YMRS and HRDS = manic or hypomanic episode  Controls: matched by age and gender, recruited in same hospital. | Cases: No unstable medical illness  Controls: no psychiatric or medical history or family history of psychiatric disorder, no medication. | ELISA | None | Serum BDNF cases < controls. The BDNF levels did not differ between medicated and drug-free BD patients. |
| Dell'Osso et al (2010) (40)  Italy | *16, 15 46.4(14.3) F 76%* | BDNF | Cases: BD type 1 depression by MINI, DSM IV, HDRS, CGI. Patients drug free or medicated. HDRS >24, CGI >4. Controls: - | No history of physical or other mental illness, no regular other medications.  Controls: no medications, no physical/mental disorder. | ELISA | Li, MS, AD, AP | BDNF cases < controls. |
| Dias et al (2009) (41)  Portugal | *65, 50 37.8(10.51)*  *F 68%* | BDNF | Cases: 17-55yrs DSM IV outpatients BD type 1 by MINI. YMRS <6, HDRS <7. Controls: 19-56yrs, acquaintances, hospital staff. No family or personal axis 1 or medical disorder. Matched for age, gender, education | Both: Organic disorder, concurrent psychiatric diagnosis, medical illness, substance use, ECT | ELISA | Li, MS, AD, AP | BDNF ns. Positive associations BDNF and illness duration, and manic and depressive episodes in female BD patients only. |
| Fernandes et al (2009) (44) Brazil | *40,10,30 41.3(8.5) F 68%* | BDNF | Cases: BD type 1 & depression by DSM IV SCID. BD in depression. HDRS.  Controls: - | Both: no drug use, ‘major’ medical illness. Controls: no medications, no DSM diagnosis, no family history of psychiatric disorder. | ELISA | MS, AP, AD | BD < depression < control p=0.001. |
| Karamustafalioglu et al (2015) (52)  Turkey | *68,30*  *34.8(10.6) F 0%* | BDNF | DSM IV BD mania, inpatients, 18-65, no medication <1 week.  Controls: - | Cases: Alcohol/drug use, another axis 1 disorder, medical disease Controls: - | ELISA | None | BDNF cases < controls. |
| Kenna et al (2014) (53) USA | *47,26 32.9(6.4) F 100%* | BDNF | 18-45, euthymic, outpatients & local community. DSM IV SCID, MADRS. Controls: recruited from adverts. | Cases: substance use, ‘uncontrolled’ medical conditions, peri-/post-menopause, COCP <3m, pregnancy/breastfeeding, endocrine disease, mood disorder sec. gen med condition, no recent <3m med change. Controls – no psychiatric diagnosis. | ELISA | - | BDNF levels n/s. Higher MADRS in BD related to lower BDNF p=0.028, p>0.05 in controls |
| Li et al (2014) (58) China* | *203,167 31.1(4.8) F 75%* | BDNF | Cases: DSM IV; HDRS >17, YMRS. First depressive episode, 19-50yrs.  Follow up diagnosis of BD disorder (manic or hypomanic episode) with SCID DSM IV. Controls: recruited by advertisement, HDRS <7. | Cases: Other axis-1 disorder, ‘severe’ medical illness, pregnancy. Controls: any axis-1, family history of ‘severe’ physical illness. | ELISA | None | BD<MDD<HC p=0.02 BD<MDD p=0.04 BD1 / BD2 p=0.28 BDNF serum BD<MDD<HC p<0.001 no difference BD/MDD p=0.05 No correlation BDNF mRNA/serum and severity p>0.05 |
| Lin et al (2016) (59)  Taiwan | *30, 30*  *30.2(5.1) F 48%* | BDNF | Cases: DSM IV BD manic episode, SCID, YMRS >26. Controls: - | Cases: Heavy tobacco use, alcohol use, substance use. Controls: no physical illness. | ELISA | Li, MS, AP | p>0.05 after adjustments |
| Loch et al (2015) (61) Brazil | *23, 28*  *28(?) F 83%* | NT-3, NT-4, NT-5 | Cases: DSM IV SCID BD I & II. 18-45yrs, HDRS >18, <3 lifetime episodes, <5 years illness duration. Controls: local adverts. | Cases: Rapid cycling, substance use, previous ECT, medical disorder. Controls: no axis 1 disorder or family psychiatric history, medical disorder. | ELISA | None | NT-3 and NT-4/5 were significantly increased in BD-depression compared to controls (p= 0.040 and 0.039) |
| Machado-Vieira et al (2007) (63) Brazil | *30, 30 26(4) F 76.7%* | BDNF | Cases: DSM IV SCID BD mania YMRS >25. Controls: age and gender matched. | Cases: Medication naïve. Control: No DSM IV illness. ‘clinical illness’. | ELISA | None | BDNF cases<controls p<0.001  Correlation between YMRS scores and BDNF r=-0.78 p<0.0-01. |
| Mackin et al (2007) (64)  UK** | *20, 14*  *48.6(10.8)* | BDNF | Cases: DSM IV BD (depression), men and post-menopausal women, stable on medication >4 weeks. Control: age and sex matched. | Cases: Previous head injury/organic disorder, serious medical condition, substance use. Control: no personal or family history of mental disorder. | ELISA | - | BDNF values were similar in both patient groups and in healthy controls (p<0.05) |
| Monteleone et al (2008) (88) Italy | *28,35,22* | BDNF | Cases: Outpatients, DSM IV SCID, HDRS YMRS (<8). BD 1 or 2 Normal ECG, bloods.  Controls: from clinical staff. | Cases: Pregnancy, COCP, alcohol, drug use. Controls: no psychiatric history, ‘major’ medical disorder, family psychiatric history. | ELISA | Li, MS,AP | BD<controls p<0.005 |
| Munkholm et al (2014) (65) NZ/Denmark* | *37,40 40.9(12.3) F 67%* | BDNF, NT-3 | Cases: 18-70yrs, DSM IV diagnosis rapid cycling BD, SCAN. Euthymic (HAMD-17 and YMRS<8), depressive (HAMD-17 >7 and YMRS<8), manic/hypomanic (YMRS>7 and  HAMD-17 <8) and mixed state (HAMD-17>7 and YMRS>7) Controls: healthy local blood donors, 18-70yrs. | Cases: ‘Significant’ physical illness, drug use, insufficient Danish skills, pregnancy.  Controls: no personal or family psychiatric illness. | ELISA | Li, MS, AD, AP | BDNF Cases>controls (p=0.001)  NT-3 p>0.05 BDNF cases>controls in euthymia (p=0.004) depression (p=0.002) mania (p=0.002), but not significant in mixed state (p>0.05) |
| Rabie et al (2014) (69) Egypt | *25,25,15,15 32.1(8.3) F 60%* | BDNF | Cases: 20-50yrs, DSM IV SCID, HAM-D, YMRS. Controls: - | Cases: Drug abuse, head trauma, medical disease. Controls: - | ELISA | - | Cases<controls p<0.01 BD dep<controls p<0.001 BD man<controls p<0.001 BD man<BD dep p=0.014 |
| Rosa et al (2014) (70) UK | 50,50 41.6(13.6) F 78% | BDNF, Oxidised Glutathione | Cases: DSM-IV SCID, ‘subsyndromal’, >16 BD type 1 Controls: matched age and sex, same geographical area. | Cases: - Controls: No psychiatric disorder | ELISA | Li, MS, AP | Cases<Controls p<0.05 |
| Suwalska et al (2010) (73) Poland | *141, 75*  *53.7(12.7)*  *F 79%* | BDNF | Cases: DSM IV SCID-I, outpatients, 30-77yrs, lithium >5 years, HRDS <7, YMRS <7. Control: age and gender matched. | Cases: Previous ECT, pregnancy. Control: no history of psychiatric or medical disorder. | ELISA | Li | lithium-treated patients lower BDNF levels compared to the healthy controls (p<0.05) |
| Tramontina et al (2009) (74)  Brazil | *10, 10*  *34.4(4.0)*  *F 50%* | BDNF | Cases: DSM IV SCID BD type 1 acute mania, inpatients. Controls: age and gender matched. | Cases: Comorbid medical illness, other medication. Control: history of psych/medical illness, organic illness. | ELISA | Li, AP | BDNF in BD-mania cases<controls mania (p<0.05) but ns after treatment (p = 0.126).  A sharp increase in BDNF level was found after treatment of the episode of acute mania (p = 0.010). |
| Tunca et al (2014) (78) Turkey | *96, 61 38.3(11.6) F 56.7%* | BDNF, GDNF | Cases: DSM IV SCID BD 1 and 2, inpatients and outpatients, CGI, YMRS, HDRS. Controls: volunteers, staff members. | Cases: Mixed/hypomanic episodes. No ‘active’ medical conditions. Controls: No medication, no history psychiatric disorders (SCID). | ELISA | Li | BDNF mania & BD depression < control, euthymia (p=0.0003, p=0.0001)  BDNF euthymia=controls (p=0.310)  BDNF mania=BD depression (p=0.782) |
| Walz et al (2009) (89)  Brazil | *154, 30*  *43.6(13.7) F 65%* | NT4/5 | Cases: DSM IV BD type 1 SCID, inpatients/outpatients Controls: hospital catchment area, matched for age and sex | Cases: Comorbid mental retardation  Control: personal or family psychiatric disorder | ELISA | Li, MS, AP, AD | NT-4/5 levels were increased in mania, depression and euthymia, but not significantly different between BD mood states |
| Yatham et al (2009) (90)  Canada | *56 56* | BDNF | Cases: Outpatients, DSM IV BD, YMRS, HDRS, Controls: matched for age, gender, education | Cases: Significant comorbid physical illness, no other medication.  Controls: Psychiatric disorder, medical disorder, organic disorder, medication. | ELISA | - | Decrease in BDNF levels accelerated with age in (cases<controls). |
| Oxidative Stress Markers | | | | | | | |
| Andreazza et al (2007) (29)  Brazil | *84,32*  *43.4(8.0)*  *F 35%* | S100b, SOD, TBARS, GPx | Cases: DSM IV SCID-I. YMRS, HDRS. >18 years, outpatients & inpatients.  Controls: matched for age and gender. | Cases: Smoking, no active medical condition, no other medication. Controls: non-smokers, ‘healthy’. | TBARS, ELISA | Li, MS | Increment of serum S100B during mania and depression, but not in euthymia. SOD increased in manic and depressed patients. TBARS  increased in BD regardless of mood phase. |
| Asdemir et al (2016) (30) Turkey | *67, 24*  *33.1(12.4)*  *F 42%* | AVP | Cases: DSM-IV criteria BD 1-2 inpatients/outpatients  Controls: - | Cases: ECT<6 months, known physical disorder, substance use, organic brain disorder.  Control: psychiatric disorder, physical disorder. | ELISA | MS, AP, AD | Lower serum AVP levels in BD-I during manic, depressive, or remission periods compared to healthy controls |
| Aydemir et al (2014) (31) Turkey | *50,51*  *40.8(11.5) F 47.1%* | GSH, SOD, NO, MDA, NT-4, | Cases: 18-65yrs, DSM IV BD in remission >6months (HDRS <7, YMRS <4). Controls: - | Cases: Axis 1 disorder, organic illness, no other medication. Control: No psychiatric or physical illness. | ELISA | - | There was no statistically significant correlation between oxidative stress markers and  cognitive functions |
| Gergerlioglu et al (2007) (46)  Turkey | *29, 30*  *33.1(9.6)*  *F 48%* | NO, SOD | Cases: DSM IV BD manic episode.  Control: ‘healthy’, from hospital staff. Matched according to smoking habits. | Both: Substance use, chronic systemic diseases, organic disorder, use of antioxidants. | Griess reaction, NBT reduction | - | NO (p<0.001) BD>controls. SOD (p<0.001) BD<controls. |
| Kapczinski et al (2011) (51) Brazil┼ | *60,80,15 40.7(12.5) F 68%* | BDNF, NT-3, TNFα, IL-6, IL-10, TRAP, TBARS, PCC | Cases: Adults, DSM-IV SCID BD type 1, inpatients & outpatients. Controls: matched age & sex from local area. | Cases: comorbid mental retardation, ‘overt’ medical illness. Controls: psychiatric comorbidity / family psychiatric history. | ELISA, TBARS method, DNPH | Li, MS, AD, AP | BD vs controls = PCC (p<0.01), TBARS (p=0.001), IL-10 (p=0.004), NT-3 (p=0.024). BDNF, TRAP, IL-6 not significant (p>0.05) BD dep vs control = PCC (p<0.001), TBARS (p<0.001), IL-10 (p<0.001), NT-3 (p=0.008) BD mania vs control = TBARS (p=<0.001), PCC (p<0.001), IL-10 (p=0.002), TNFα (p=0.006)  BD euthymia vs control = NT-3 (p=0.003), IL-10 (p=0.009) |
| Selek et al (2008) (71)  Turkey | *30,30*  *32.8(12.2)*  *F 42%* | SOD, NO | DSM IV BD type 1 (depression), outpatients. Control: Hospital staff, matched for age, sex and smoking status. | Cases: Substance use, chronic systemic diseases, organic brain disorder Controls: - | NBT reduction | MS, AD, AP | NO BD>controls/ SOD BD<controls. NO BD<controls (all p<0.05). |
| Versace et al (2014) (80)  USA | *24, 19*  *33.1(8.3)*  *F 66%* | LPH, 4-HNE | Cases: DSM IV BD I (euthymia) remission >2 months, HDRS <18, YMRS <10.  Controls: - | Cases: - Controls: No psychiatric illness. | Not stated | MS, AD, AP | significant difference BD vs controls in LPH  p=0.022), but not 4-HNE. |
| Yanik et al (2004) (82)  Turkey | *43, 31* | Arginine, Manganese, NO | Cases: DSM IV BD I – Mania.  Controls: Age, gender & smoking status matched. | Both: Substance use, chronic systemic disease, organic brain disorder, ECT. | Griess reaction | MS, AD, AP | Plasma arginase activities and Manganese were  found to be significantly lower and total nitrite level higher in patients with BD compared with controls |

Notes: BD – bipolar disorder, MDD – Major depressive disorder, MS – mood stabilisers, Li – lithium, AP – antipsychotics, AD – antidepressants, hsCRP – high sensitivity C-reactive protein, IFN-γ – interferon-gamma, IL1-RA – interleukin 1 receptor antagonist, IL1-β – interleukin 1-β, IL-2 – interleukin 2, sIL-2R – soluble IL-2 receptor, IL-4 – interleukin 4, IL-5 – interleukin 5, IL-6 – interleukin 6, sIL-6R – soluble IL-6 receptor, IL-8 – interleukin 8, IL-10 – interleukin 10, IL-12 – interleukin 12, IL-17 – interleukin 17, IL-18 – interleukin 18, IL-18BP – IL-18 binding protein, IL-22 – interleukin 22, TNF-α – tumour necrosis factor alpha, sTNFR1 – soluble TNF-α receptor 1, sTNFR2 – soluble TNF-α receptor 2, TGF-β1 – transforming growth factor beta-1, sP-selectin – soluble P-selectin, MCP-1 – monocyte chemotactic protein-1, FGF-β – fibroblast growth factor-β, VEGF – vascular endothelial growth factor, BDNF – brain derived neurotrophic factor, NT-3 – neurotrophin 3, NT-4/5 – neurotrophin 4/5, GDNF – glial cell line-derived neurotrophic factor, PCC – protein carbonyl content, TBARS – thiobarbituric acid reactive substances, SOD – superoxide dismutase, GSH – total glutathione, GPx – glutathione peroxidase activity, AVP – arginine vasopressin, NO – nitric oxide, MDA – malondialdehyde, TRAP – total reactive antioxidant potential, LPH – lipid hydroperoxides, 4-HNE – 4-hydroxy-2-nonenal, DSM – Diagnostic and Statistical Manual of Mental Disorders, ICD – International Classification of Diseases, SCID – Structured Clinical Interview for DSM-IV, M.I.N.I – Mini-international Neuropsychiatric Interview, SCAN – Schedules for Clinical Assessment in Neuropsychiatry, PDA – Psychiatric Diagnostic Assessment, HDRS/HAM-D – Hamilton Rating Scale for Depression, MADRS – Montgomery-Asberg Depression Rating Scale, IDS - Inventory of Depressive Symptomatology, YMRS – Young Mania Rating Scale, CGI – Clinical Global Impression, GAF- Global Assessment of Functioning, BPRS – Brief Psychiatric Rating Scale, FBC – Full Blood Count, U&E – Urea and Electrolytes, TFT – Thyroid Function Tests, LFT – Liver Function Tests, CXR – Chest X-ray, ECG - Electrocardiogram, EEG – Electroencephalogram, BMI – Body Mass Index, VDRL – Venereal Disease Research Laboratory Test, ELISA – enzyme-linked immunosorbent assay, CBA – cytometric bead array, NBT – nitroblue tetrazolium, CS – cellular supernatents, PEI - Particle enhanced immunonephelometry, CSRI - Classical single radial immunodiffusion, PEN - particle-enhanced nephelometry
┼Study measures more than one class of biomarker
**All studies case control except
*cohort
**cross-over trial**
