# Supplementary material for: Neurotrophins, cytokines, oxidative stress mediators and mood state in bipolar disorder: systematic review and meta-analyses
Source: Br J Psychiatry. 2018 Sep;213(3):514–25. doi: 10.1192/bjp.2018.144 (PMC6429261; doi:10.1192/bjp.2018.144)
Supplement: Supplementary file 1 [file S0007125018001447sup001.zip › S0007125018001447sup001/Supplementary data Table 2 - Newcastle Ottawa Scales for included studies.docx]

Supplementary Table 2: Newcastle-Ottawa Scale assessment of studies

|  | Selection | | | | Comparability | Exposure | | | Total |
| --- | --- | --- | --- | --- | --- | --- | --- | --- | --- |
| **Author/year** | **1** | **2** | **3** | **4** | **(max 2)** | **1** | **2** | **3** | **/9** |
| **Inflammatory markers** |  |  |  |  |  |  |  |  |  |
| Bai et al (2014) | 0 | 0 | 0 | 1 | 1 | 0 | 0 | 1 | 3 |
| Barbosa et al (2011) | 1 | 0 | 0 | 0 | 2 | 0 | 1 | 0 | 4 |
| Barbosa et al (2012) | 1 | 0 | 0 | 0 | 2 | 0 | 1 | 0 | 4 |
| Breunis et al (2003) | 1 | 0 | 1 | 1 | 2 | 0 | 1 | 0 | 6 |
| Brietzke et al (2009) | 1 | 0 | 0 | 1 | 2 | 0 | 0 | 0 | 4 |
| Cetin et al (2012) | 1 | 0 | 1 | 1 | 2 | 0 | 0 | 0 | 5 |
| De Berardis et al (2008) | 0 | 0 | 0 | 1 | 2 | 0 | 0 | 0 | 3 |
| Do Prado et al (2013) | 1 | 1 | 0 | 1 | 2 | 0 | 1 | 0 | 6 |
| Drexhage et al (2011) | 1 | 0 | 1 | 1 | 1 | 0 | 1 | 0 | 5 |
| Fiedorowicz et al (2015) | 1 | 0 | 1 | 1 | 2 | 0 | 1 | 0 | 6 |
| Guloksuz et al (2010) | 1 | 1 | 1 | 1 | 1 | 0 | 0 | 0 | 5 |
| Huang et al (2007) | 1 | 1 | 0 | 0 | 0 | 1 | 0 | 0 | 3 |
| Hope et al (2011) | 1 | 0 | 1 | 1 | 1 | 1 | 0 | 0 | 5 |
| Jacoby et al (2016) | 1 | 1 | 1 | 1 | 0 | 0 | 1 | 0 | 5 |
| Kauer-Sant'Anna et al (2009) | 1 | 1 | 0 | 1 | 2 | 0 | 1 | 0 | 6 |
| Kim Y et al (2002) | 1 | 1 | 1 | 1 | 0 | 1 | 1 | 0 | 6 |
| Kim Y et al (2007) | 1 | 1 | 1 | 1 | 0 | 0 | 0 | 0 | 4 |
| Kim Y et al (2004) | 1 | 1 | 1 | 1 | 0 | 0 | 0 | 1 | 5 |
| Kunz et al (2011) | 1 | 1 | 1 | 1 | 0 | 1 | 1 | 0 | 6 |
| Liu et al (2004) | 1 | 1 | 1 | 1 | 1 | 1 | 1 | 0 | 7 |
| Legros et al (1985) | 0 | 1 | 0 | 0 | 0 | 1 | 0 | 0 | 2 |
| Lotrich et al (2014) | 1 | 1 | 1 | 0 | 0 | 1 | 1 | 0 | 5 |
| O'Brien et al (2006) | 1 | 1 | 0 | 0 | 0 | 0 | 0 | 0 | 2 |
| Ortez-Dominguez et al (2007) | 1 | 1 | 0 | 1 | 0 | 0 | 1 | 0 | 4 |
| Panizzutti et al (2015) | 1 | 1 | 0 | 0 | 0 | 0 | 1 | 0 | 3 |
| Su et al (2011) | 1 | 1 | 1 | 1 | 2 | 1 | 1 | 0 | 8 |
| Tsai et al (1999) | 1 | 0 | 1 | 1 | 1 | 1 | 0 | 0 | 5 |
| Tsai et al (2001) | 1 | 0 | 1 | 1 | 1 | 1 | 0 | 0 | 5 |
| Tsai et al (2012) | 1 | 0 | 1 | 1 | 1 | 1 | 0 | 0 | 5 |
| Uyanik et al (2015) | 0 | 0 | 0 | 1 | 0 | 1 | 0 | 0 | 2 |
| Wadee et al (2002) | 1 | 1 | 1 | 1 | 1 | 1 | 1 | 0 | 7 |
| **Neurotrophins** |  |  |  |  |  |  |  |  |  |
| Barbosa et al 2013 | 1 | 1 | 1 | 1 | 2 | 1 | 1 | 0 | 8 |
| Barbosa et al 2014 | 1 | 0 | 1 | 1 | 2 | 1 | 1 | 0 | 7 |
| Cunha et al 2006 | 1 | 0 | 0 | 1 | 2 | 1 | 1 | 0 | 6 |
| De Oliveira et al 2009 | 1 | 0 | 1 | 1 | 0 | 1 | 1 | 0 | 5 |
| Dell'Osso et al 2010 | 1 | 0 | 0 | 1 | 0 | 1 | 1 | 0 | 4 |
| Dias et al 2009 | 1 | 0 | 1 | 1 | 0 | 1 | 1 | 0 | 5 |
| Fernandes et al 2009 | 1 | 0 | 0 | 1 | 0 | 1 | 1 | 0 | 4 |
| Karamustafalioglu et al 2015 | 0 | 1 | 0 | 0 | 2 | 1 | 1 | 0 | 5 |
| Kenna et al 2014 | 1 | 1 | 1 | 1 | 0 | 1 | 1 | 0 | 6 |
| Li et al 2014 | 1 | 1 | 1 | 1 | 2 | 1 | 1 | 0 | 8 |
| Lin et al 2016 | 1 | 1 | 0 | 1 | 0 | 1 | 1 | 0 | 5 |
| Loch et al 2015 | 1 | 1 | 1 | 1 | 0 | 1 | 1 | 0 | 6 |
| Machado-Vieira et al 2007 | 1 | 0 | 0 | 1 | 2 | 1 | 1 | 0 | 6 |
| Mackin et al 2007 | 0 | 0 | 0 | 1 | 2 | 1 | 1 | 0 | 5 |
| Monteleone et al 2008 | 1 | 1 | 0 | 1 | 0 | 1 | 1 | 0 | 5 |
| Munkholm et al 2014 | 0 | 0 | 0 | 1 | 0 | 1 | 1 | 0 | 3 |
| Rabie et al 2014 | 1 | 0 | 0 | 0 | 0 | 1 | 1 | 0 | 3 |
| Rosa et al 2014 | 1 | 1 | 1 | 1 | 2 | 1 | 1 | 0 | 8 |
| Suwalska et al 2010 | 1 | 1 | 0 | 1 | 2 | 1 | 1 | 0 | 7 |
| Tramontina et al 2009 | 1 | 0 | 0 | 1 | 2 | 1 | 1 | 0 | 6 |
| Tunca et al 2014 | 1 | 0 | 0 | 1 | 0 | 1 | 1 | 0 | 4 |
| Walz et al 2009 | 1 | 0 | 1 | 1 | 2 | 1 | 1 | 0 | 7 |
| Yatham et al 2009 | 0 | 1 | 0 | 1 | 2 | 1 | 1 | 0 | 6 |
| **Oxidative Stress** |  |  |  |  |  |  |  |  |  |
| Andreazza et al 2007 | 1 | 1 | 0 | 1 | 2 | 1 | 1 | 0 | 7 |
| Asdemir et al 2016 | 1 | 0 | 0 | 1 | 0 | 1 | 1 | 0 | 4 |
| Aydemir et al 2014 | 1 | 1 | 0 | 1 | 0 | 1 | 1 | 0 | 5 |
| Gergerlioglu et al 2007 | 0 | 0 | 0 | 1 | 1 | 1 | 1 | 0 | 4 |
| Kapczinski et al 2011 | 1 | 1 | 1 | 1 | 2 | 1 | 1 | 0 | 8 |
| Selek et al 2008 | 0 | 1 | 0 | 1 | 0 | 1 | 1 | 0 | 4 |
| Versace et al 2014 | 1 | 0 | 0 | 1 | 2 | 1 | 1 | 0 | 6 |
| Yanik et al 2004 | 1 | 0 | 0 | 0 | 2 | 1 | 1 | 0 | 5 |

Scale items - Selection: 1. Is the case definition adequate? 2. Representativeness of the cases. 3. Selection of controls. 4. Definition of controls. Comparability: Comparability of cases and controls on the basis of design or analysis. Exposure: 1. Ascertainment of exposure. 2. Same method of ascertainment for cases and controls. 3. Non-response rate.
