# Supplementary figures and images for: Neurotrophins, cytokines, oxidative stress mediators and mood state in bipolar disorder: systematic review and meta-analyses
Source: Br J Psychiatry. 2018 Sep;213(3):514–25. doi: 10.1192/bjp.2018.144 (PMC6429261; doi:10.1192/bjp.2018.144)

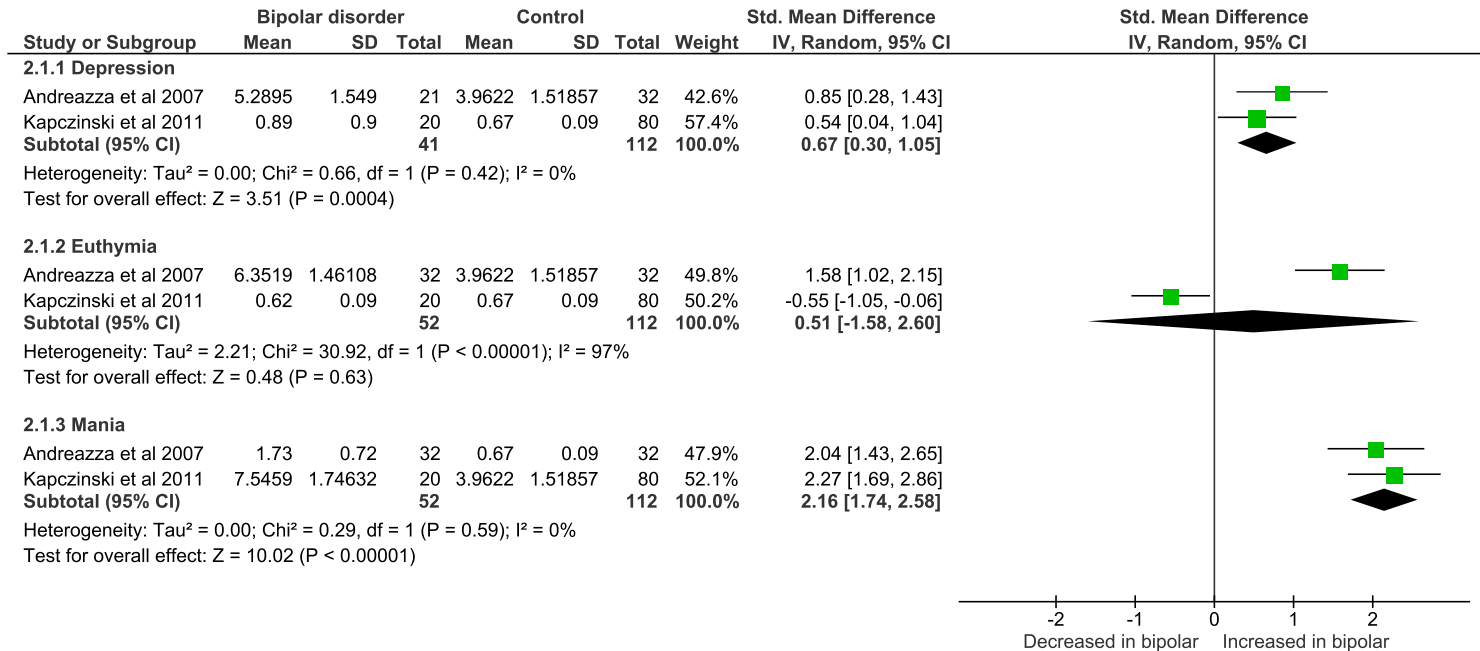

Supplement: Supplementary file 1 [file S0007125018001447sup001.zip › S0007125018001447sup001/Supplementary data Figure 10 - Forest plot - TBARS.pdf]

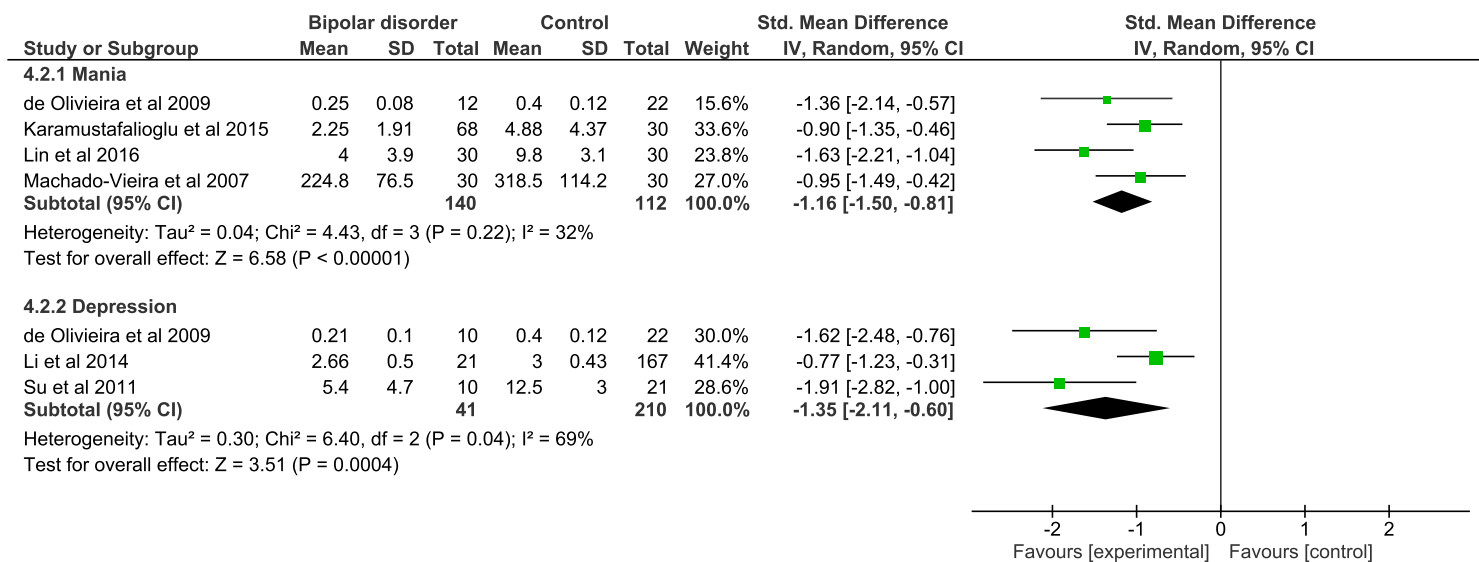

Supplement: Supplementary file 1 [file S0007125018001447sup001.zip › S0007125018001447sup001/Supplementary data Figure 11 - Forest plot - BDNF medication free.pdf]

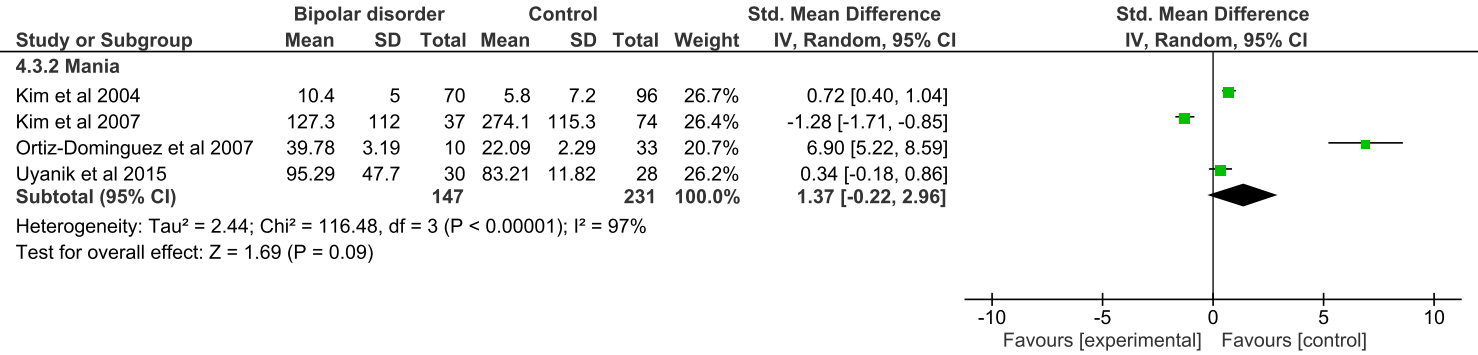

Supplement: Supplementary file 1 [file S0007125018001447sup001.zip › S0007125018001447sup001/Supplementary data Figure 12 - Forest plot - IL-4 medication free.pdf]

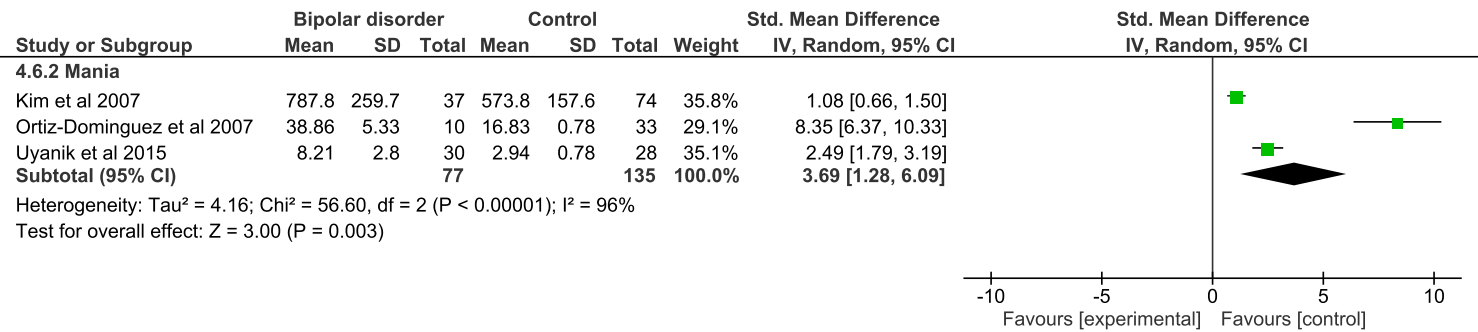

Supplement: Supplementary file 1 [file S0007125018001447sup001.zip › S0007125018001447sup001/Supplementary data Figure 13 - Forest plot - TNF-+▌ medication free.pdf]

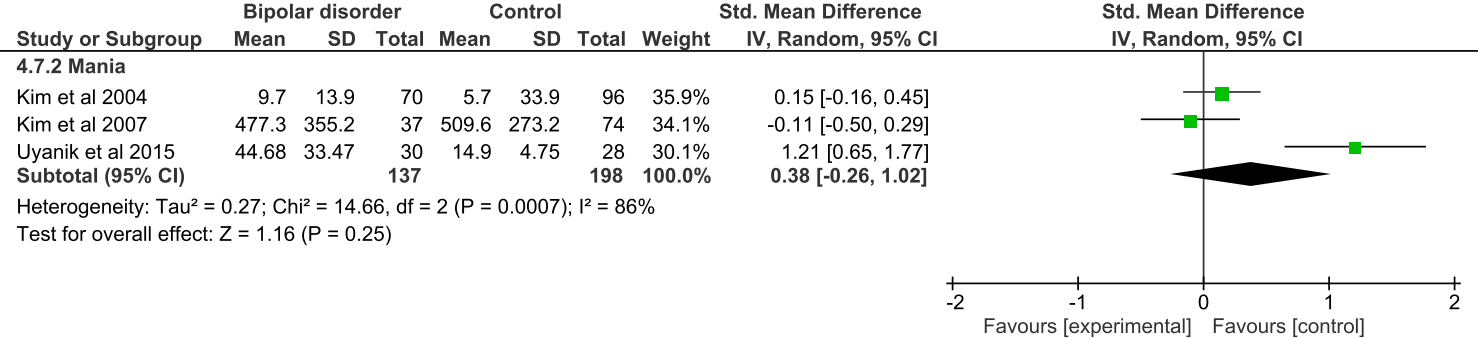

Supplement: Supplementary file 1 [file S0007125018001447sup001.zip › S0007125018001447sup001/Supplementary data Figure 14 - Forest plot - IFN-+▌ medication free.pdf]

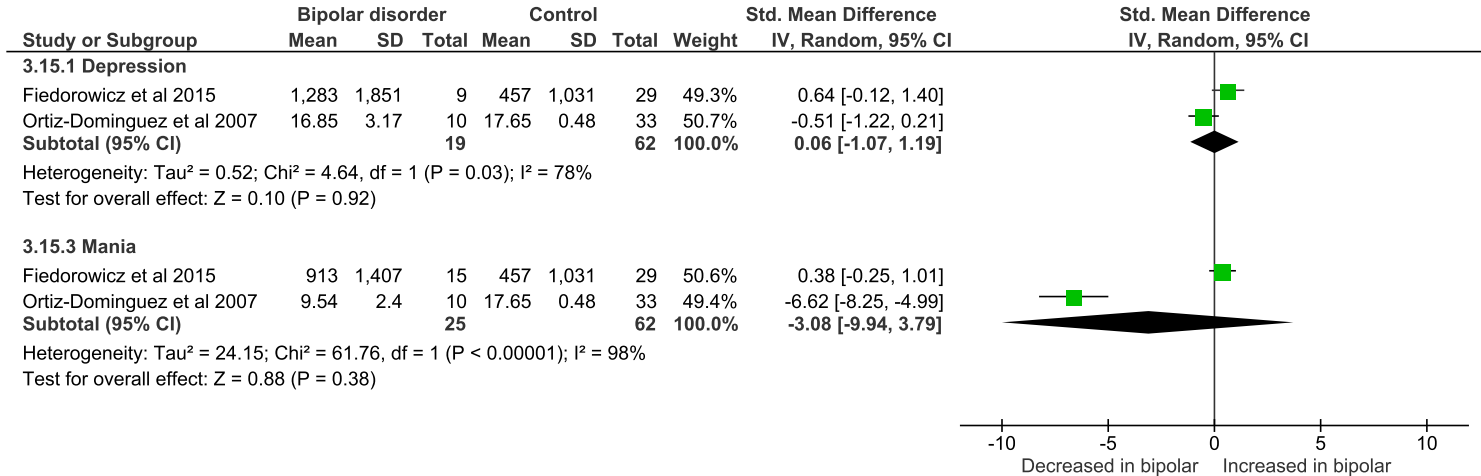

Supplement: Supplementary file 1 [file S0007125018001447sup001.zip › S0007125018001447sup001/Supplementary data Figure 15 - Forest plot - IL1-+▌.pdf]

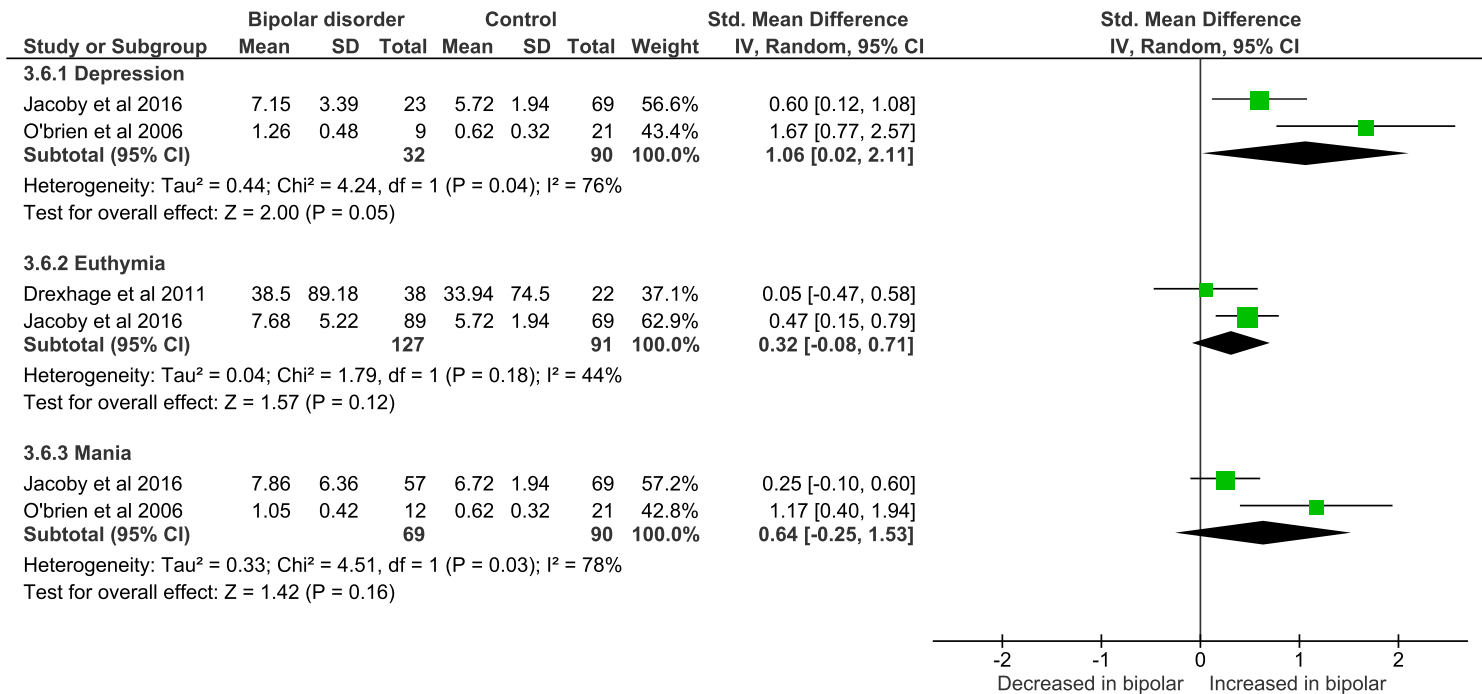

Supplement: Supplementary file 1 [file S0007125018001447sup001.zip › S0007125018001447sup001/Supplementary data Figure 16 - Forest plot - IL-8.pdf]

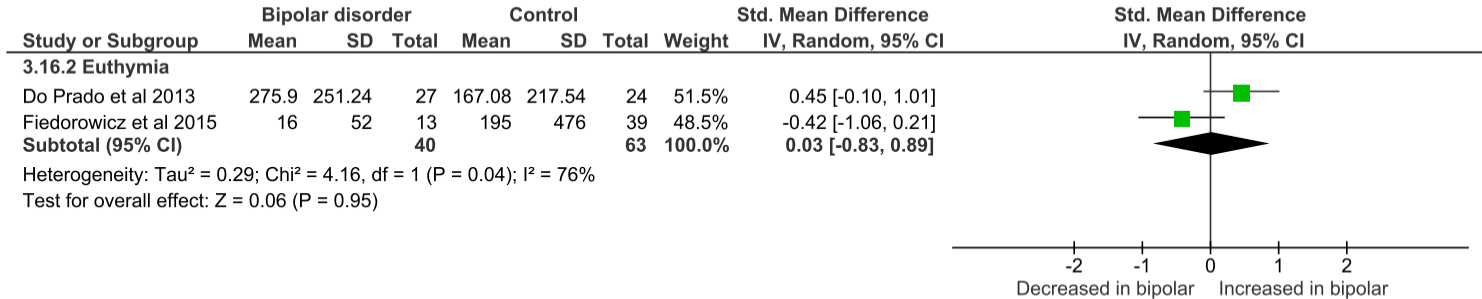

Supplement: Supplementary file 1 [file S0007125018001447sup001.zip › S0007125018001447sup001/Supplementary data Figure 17 - Forest plot - IL-17.pdf]

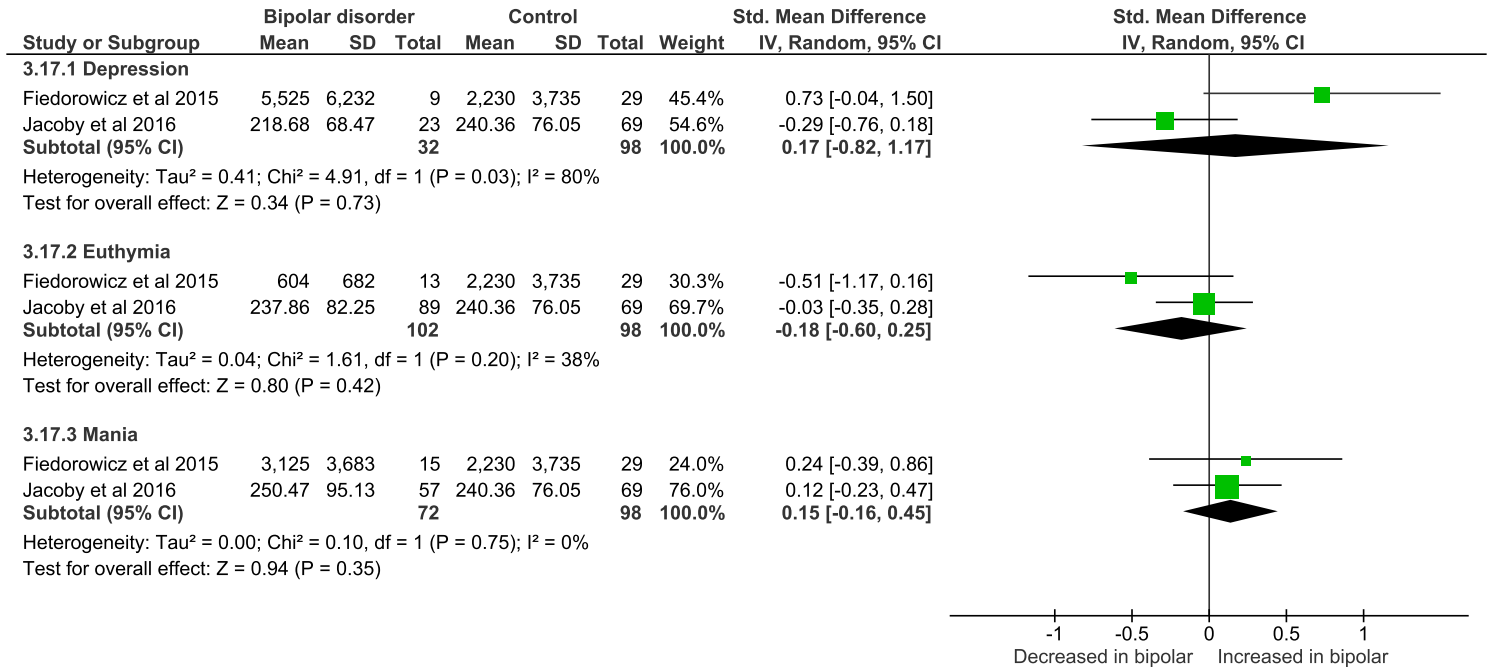

Supplement: Supplementary file 1 [file S0007125018001447sup001.zip › S0007125018001447sup001/Supplementary data Figure 18 - Forest plot - IL-18.pdf]

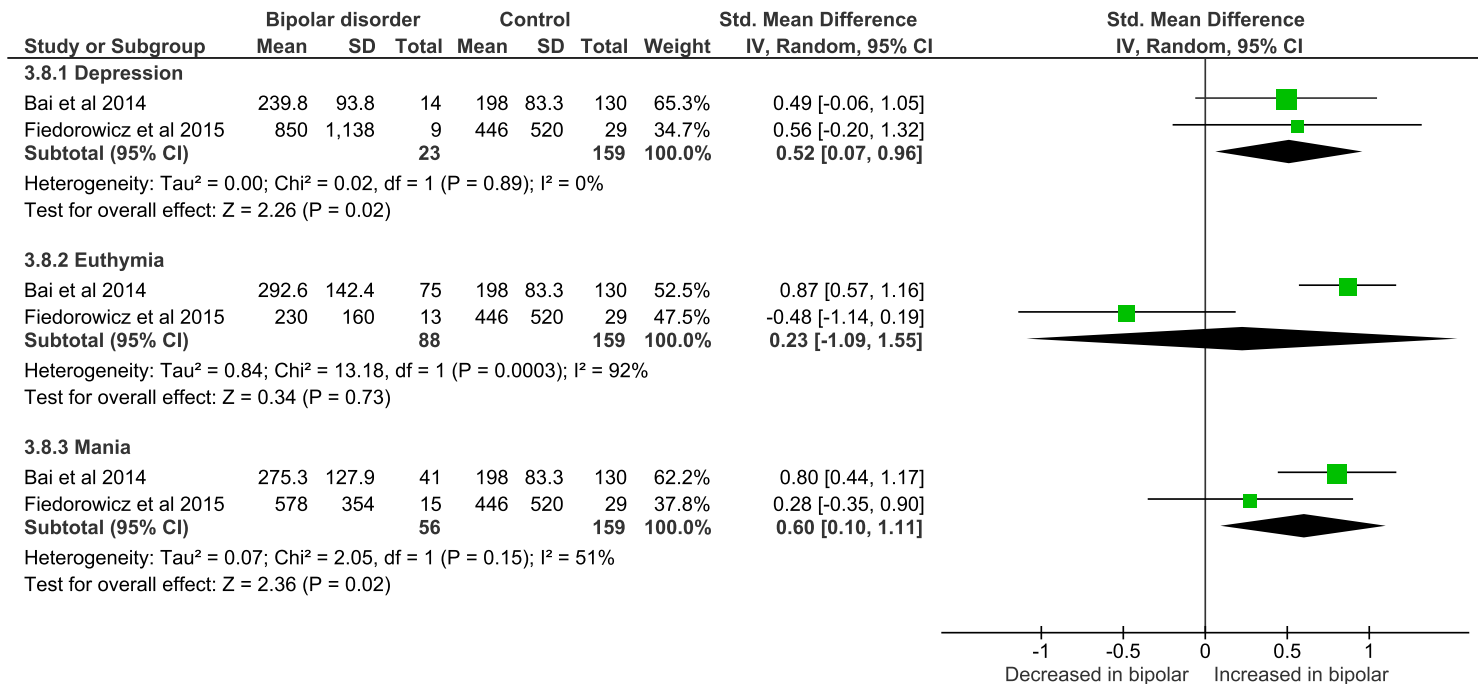

Supplement: Supplementary file 1 [file S0007125018001447sup001.zip › S0007125018001447sup001/Supplementary data Figure 19 - Forest plot - MCP-1.pdf]

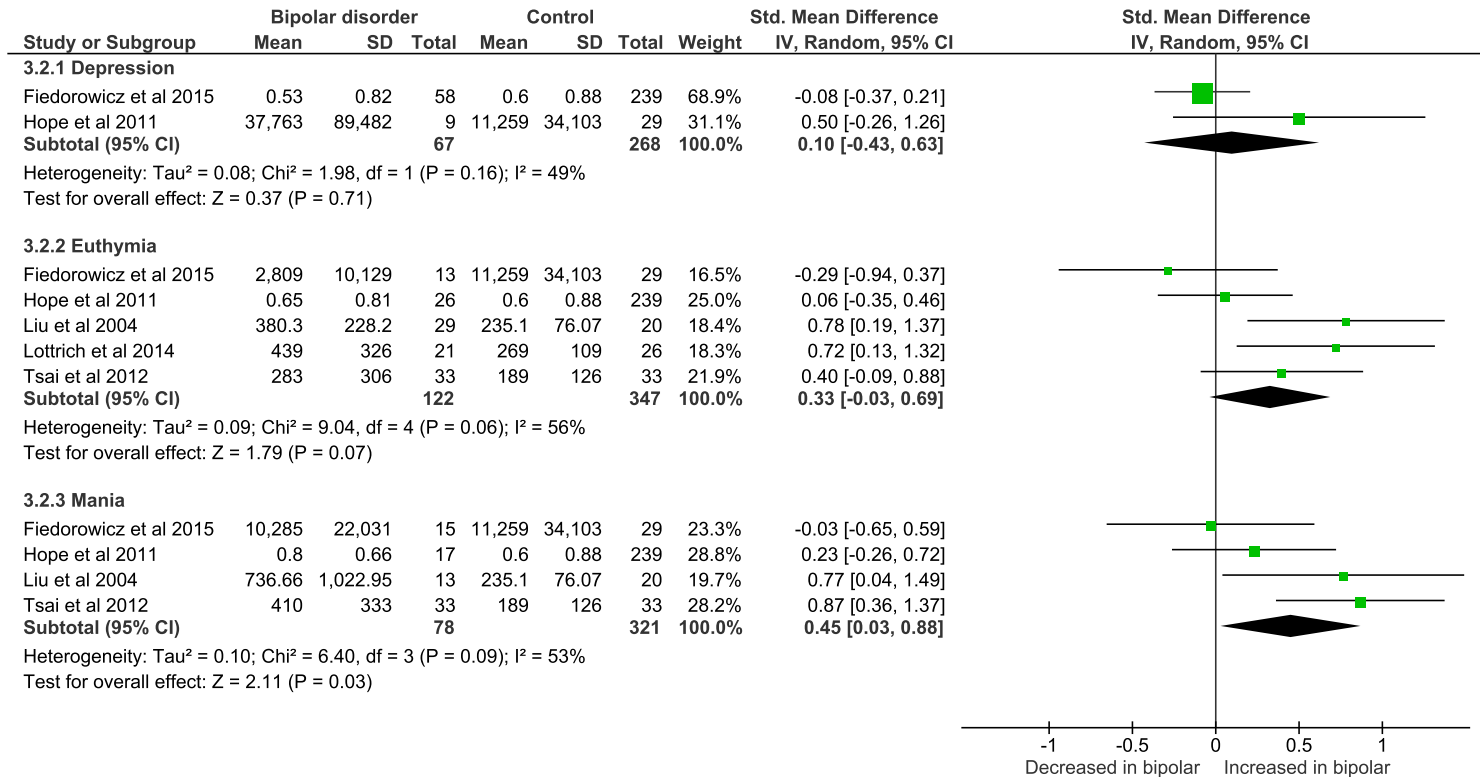

Supplement: Supplementary file 1 [file S0007125018001447sup001.zip › S0007125018001447sup001/Supplementary data Figure 2 - Forest plot - IL1-RA.pdf]

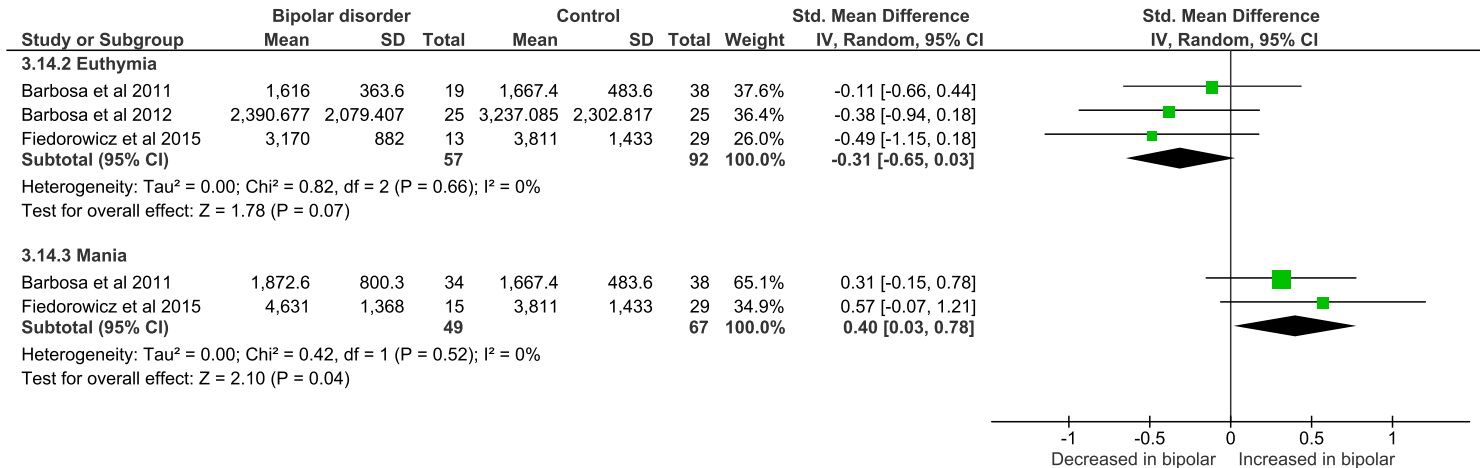

Supplement: Supplementary file 1 [file S0007125018001447sup001.zip › S0007125018001447sup001/Supplementary data Figure 20 - Forest plot - sTNFR2.pdf]

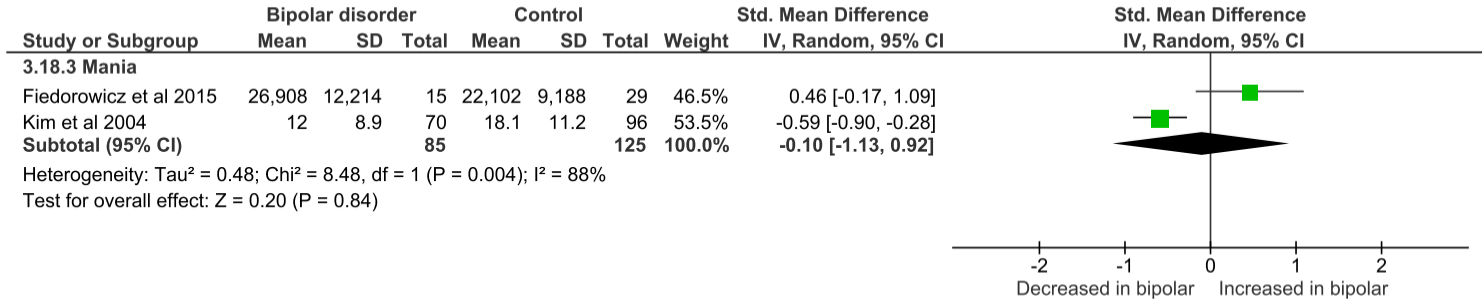

Supplement: Supplementary file 1 [file S0007125018001447sup001.zip › S0007125018001447sup001/Supplementary data Figure 21 - Forest plot - TGF-+▌1.pdf]

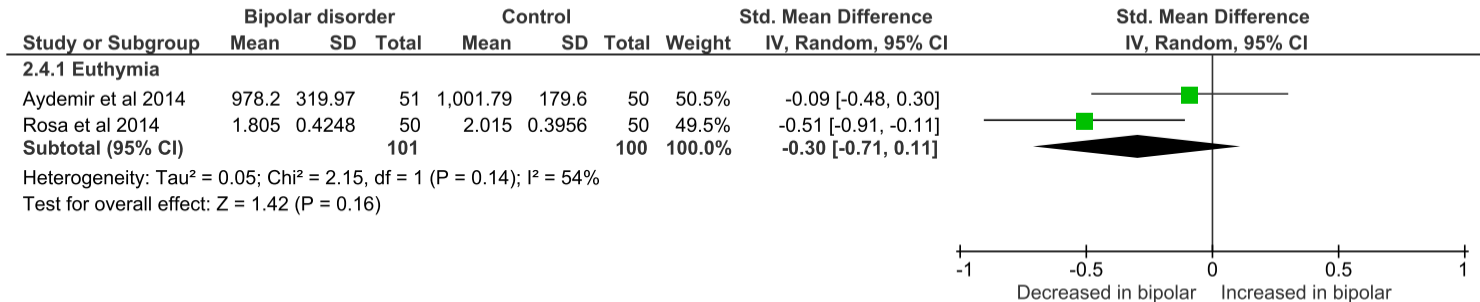

Supplement: Supplementary file 1 [file S0007125018001447sup001.zip › S0007125018001447sup001/Supplementary data Figure 22 - Forest plot - Glutathione.pdf]

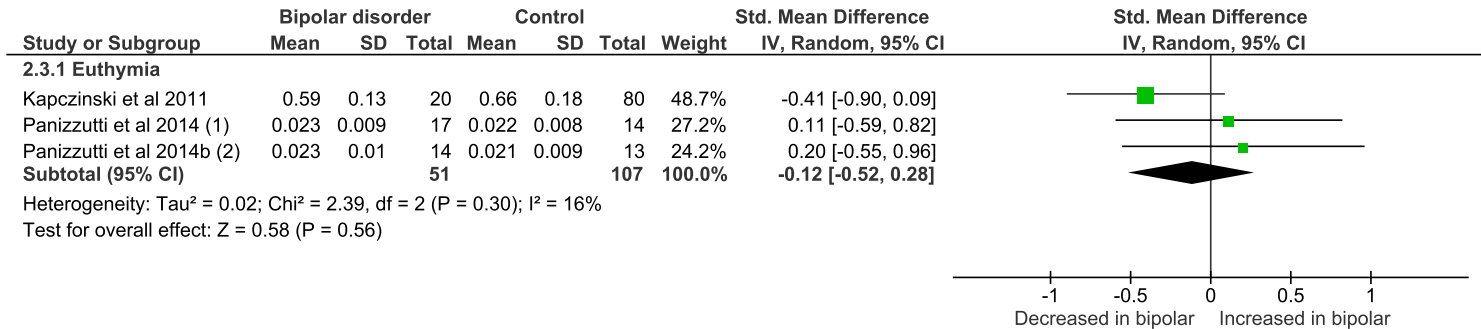

#### Footnotes

(1) Early stage

(2) Late stage

Supplement: Supplementary file 1 [file S0007125018001447sup001.zip › S0007125018001447sup001/Supplementary data Figure 23 - Forest plot - PCC.pdf]

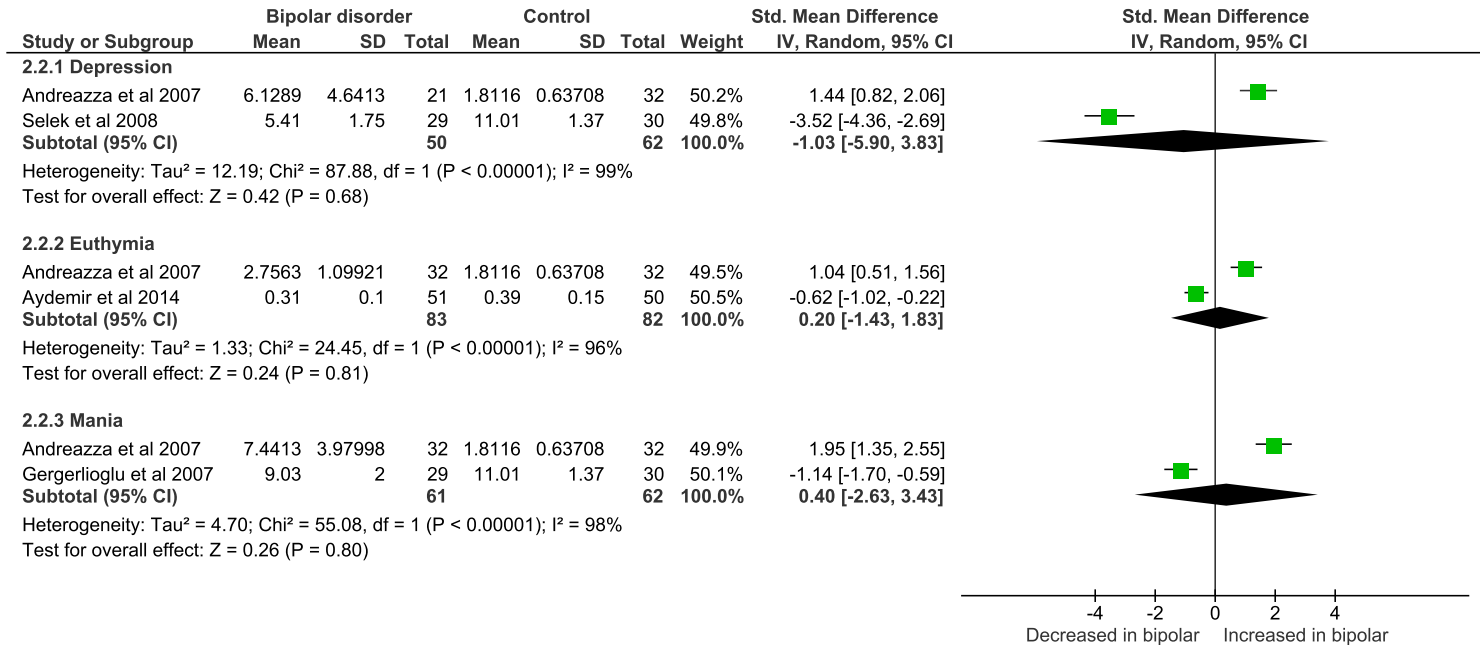

Supplement: Supplementary file 1 [file S0007125018001447sup001.zip › S0007125018001447sup001/Supplementary data Figure 24 - Forest plot - SOD.pdf]

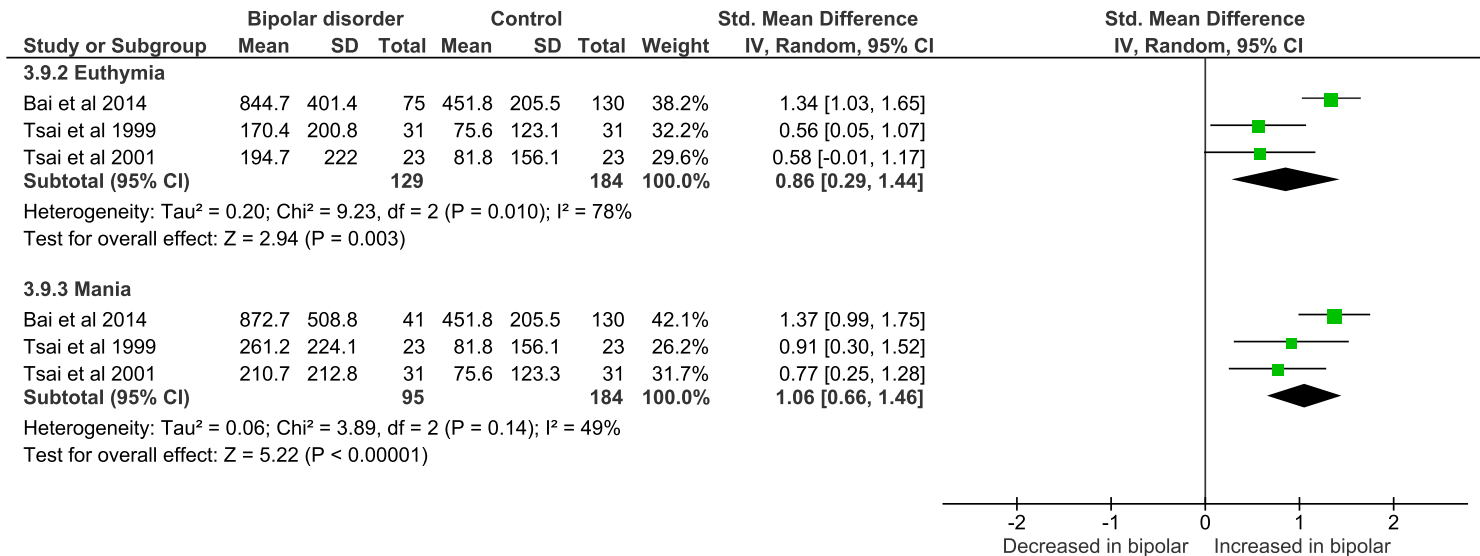

Supplement: Supplementary file 1 [file S0007125018001447sup001.zip › S0007125018001447sup001/Supplementary data Figure 3 - Forest plot - sIL-2R.pdf]

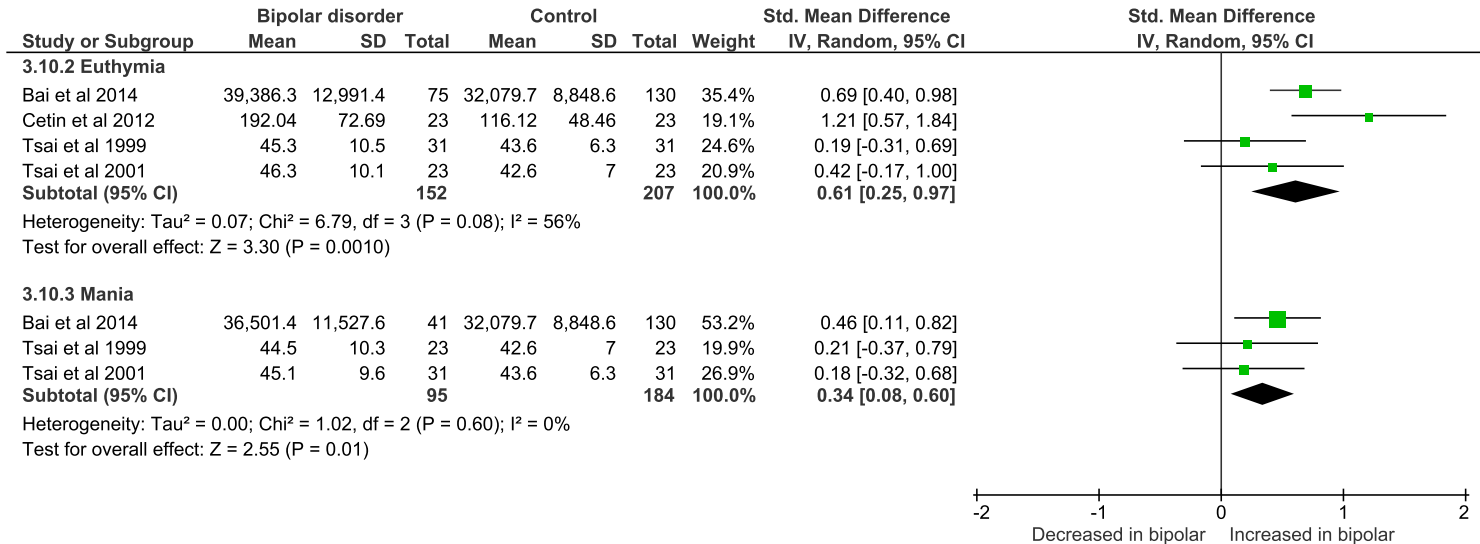

Supplement: Supplementary file 1 [file S0007125018001447sup001.zip › S0007125018001447sup001/Supplementary data Figure 4 - Forest plot - sIL-6R.pdf]

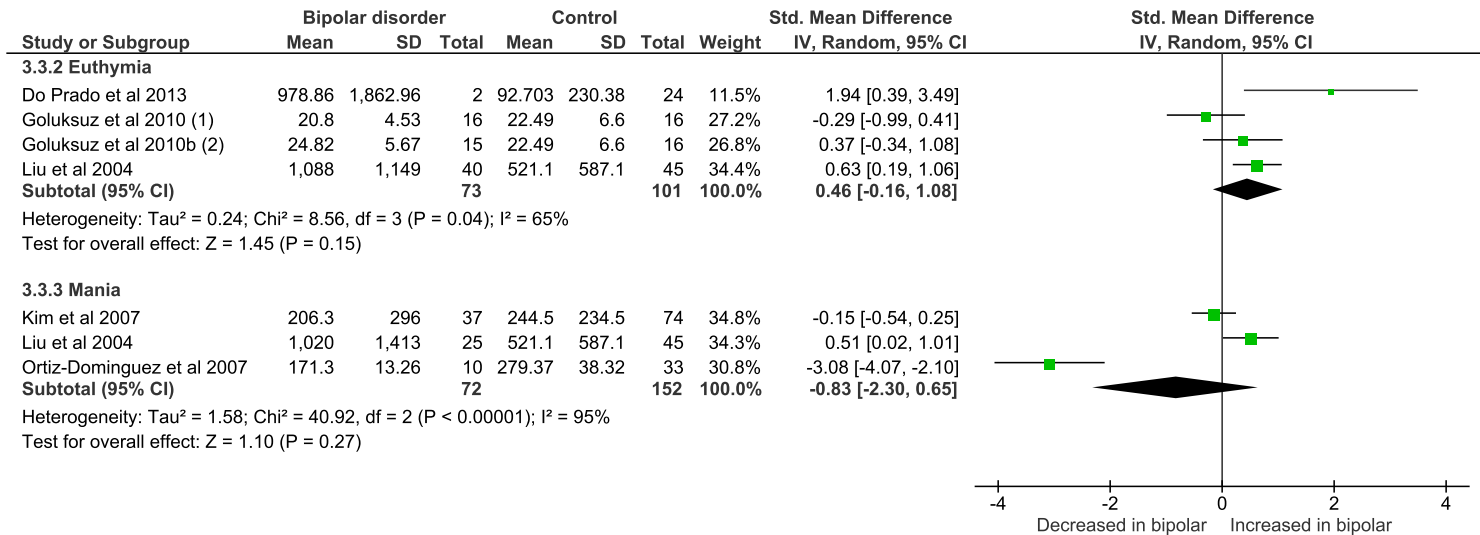

#### Footnotes

(1) Medication free

(2) lithium monotherapy

Supplement: Supplementary file 1 [file S0007125018001447sup001.zip › S0007125018001447sup001/Supplementary data Figure 5 - Forest plot - IL-2.pdf]

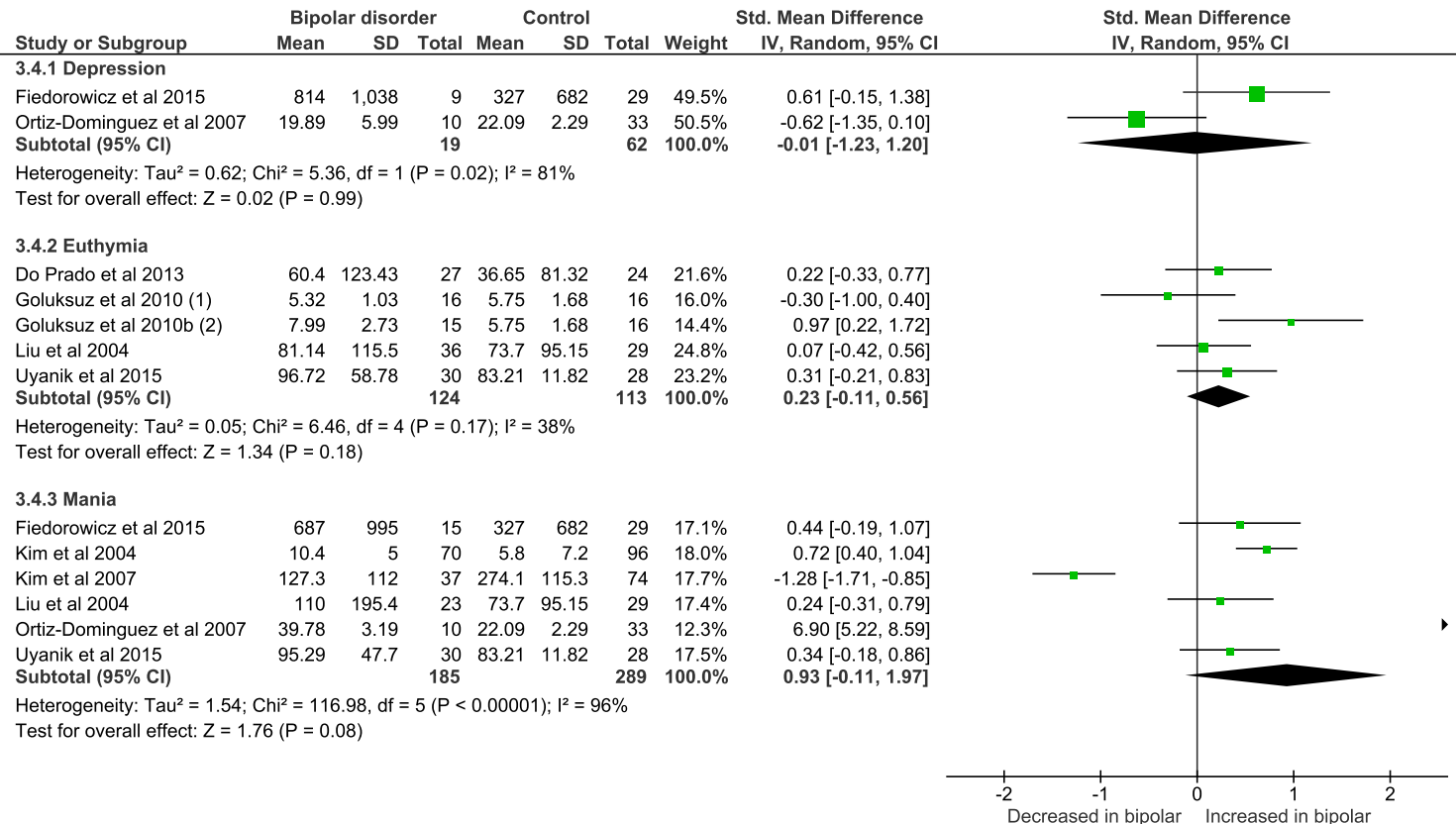

#### Footnotes

(1) Medication free

(2) Lithium monotherapy

Supplement: Supplementary file 1 [file S0007125018001447sup001.zip › S0007125018001447sup001/Supplementary data Figure 6 - Forest plot - IL-4.pdf]

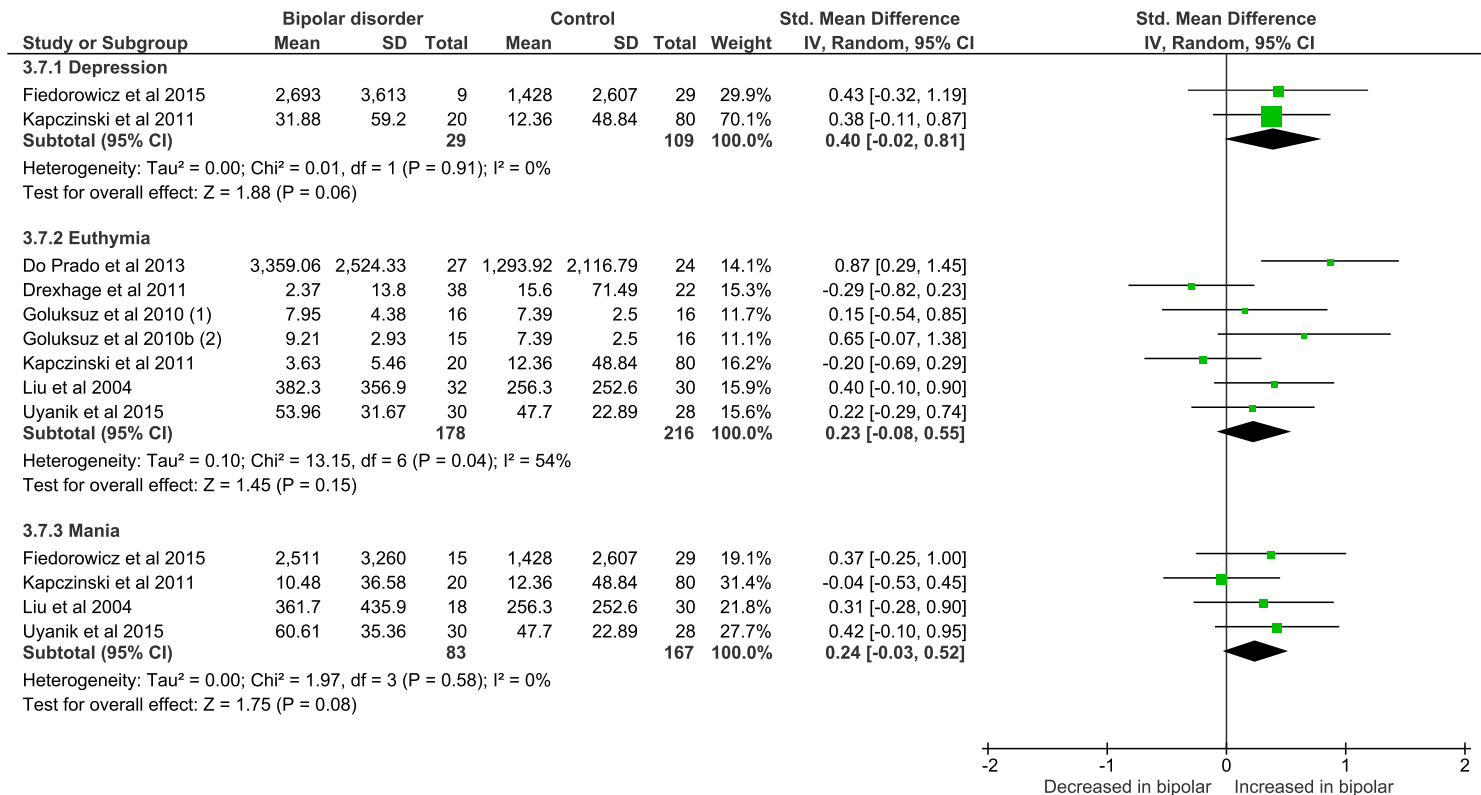

#### Footnotes

(1) Medication free

(2) Lithium monotherapy

Supplement: Supplementary file 1 [file S0007125018001447sup001.zip › S0007125018001447sup001/Supplementary data Figure 7 - Forest plot - IL-10.pdf]

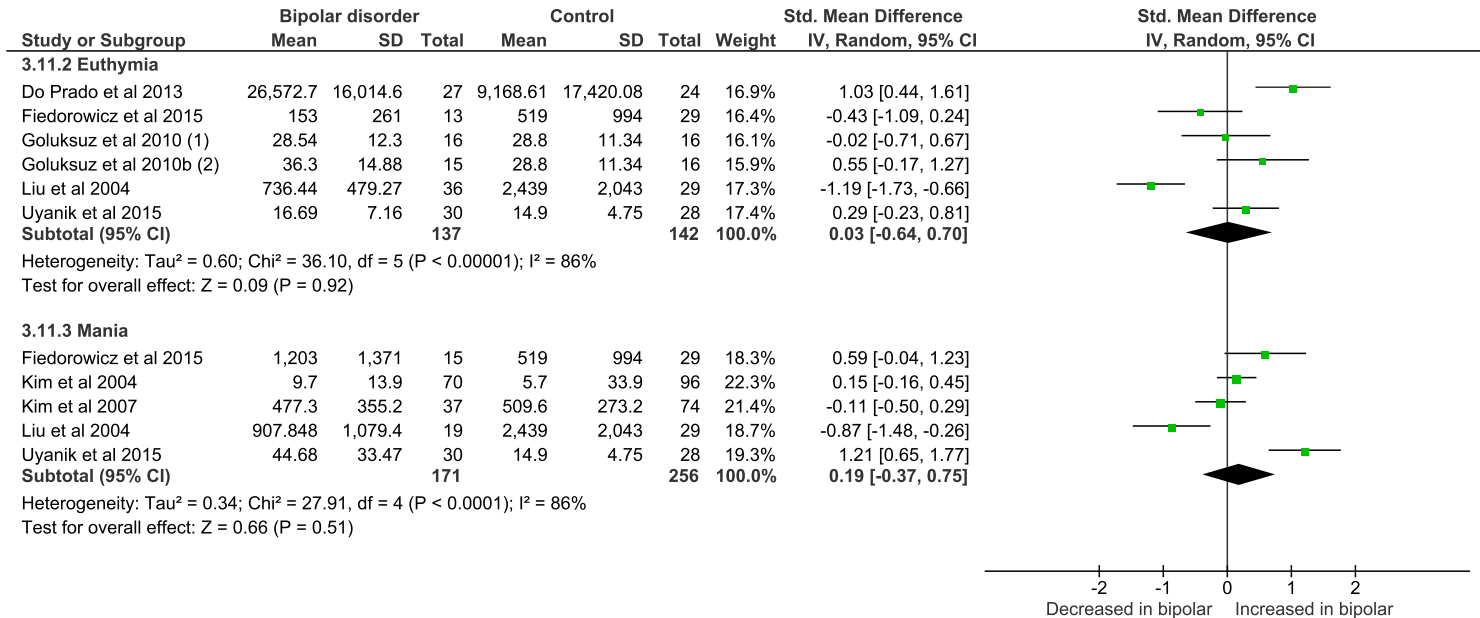

#### Footnotes

(1) Medication free

(2) Lithium monotherapy

Supplement: Supplementary file 1 [file S0007125018001447sup001.zip › S0007125018001447sup001/Supplementary data Figure 8 - Forest plot - IFN-+▌.pdf]

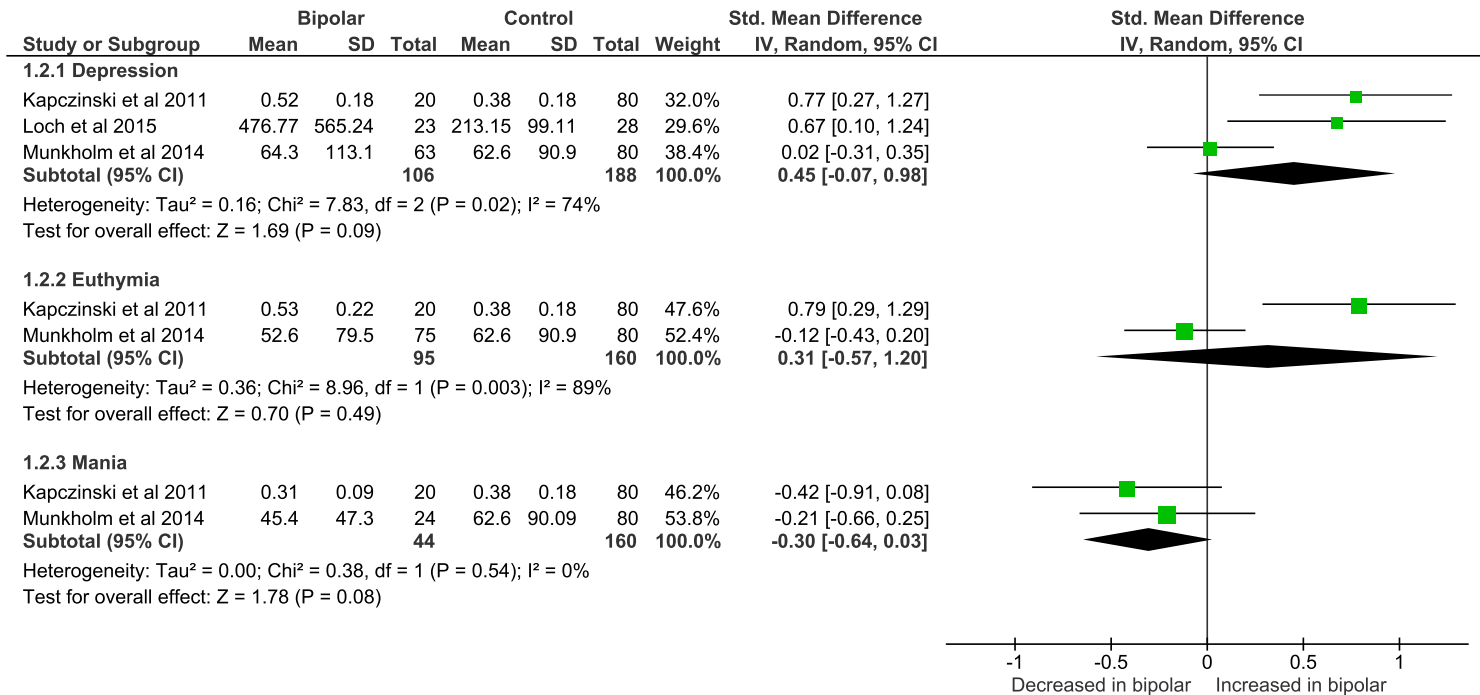

Supplement: Supplementary file 1 [file S0007125018001447sup001.zip › S0007125018001447sup001/Supplementary data Figure 9 - Forest plot - NT-3.pdf]
